# Leigh Syndrome Pathomechanism Involves Region-Specific Innate Immune Activation in *Ndufs4* Knockout Mice

**DOI:** 10.1007/s10571-026-01681-2

**Published:** 2026-02-04

**Authors:** Belinda R. Fouché, Sibonelo G. Khumalo, Werner J. H. Koopman, Marianne Venter

**Affiliations:** 1 Biomedical and Molecular Metabolism Research (BioMMet), North-West University, Potchefstroom, South Africa; 2 Department of Pediatrics, Radboud Center for Mitochondrial Medicine (RCMM), Amalia Children’s Hospital, Radboud University Medical Center (Radboudumc), Nijmegen, The Netherlands; 3 Human and Animal Physiology, Wageningen University, Wageningen, GE The Netherlands

**Keywords:** *Ndufs4* knockout mouse model, Transcriptomics, Neuroinflammation, RIG-I like signalling, Olfactory bulb

## Abstract

Although recent evidence suggests that the immune system contributes to the pathogenesis of paediatric Leigh syndrome, detailed mechanistic insights are still lacking. Here, we investigated the involvement of immune system activation and inflammation in brain tissue in Leigh syndrome, using hypothesis generating methods. We compared the transcriptomes of olfactory bulb and cerebellum from male *Ndufs4*^*−/−*^ (knockout) mice (*n* = 5–6), a well-established model of paediatric Leigh syndrome. Relative to wildtype animals, knockout mice displayed enrichment of innate immune system pathways in the olfactory bulb. Unexpectedly, relative to the olfactory bulb, few pathways were enriched in the cerebellum, and none that indicated similar changes to the immune system. Innate immune system pathways in the olfactory bulb were mainly upregulated and included a large set of interferon stimulated genes, and genes involved in JAK-STAT and retinoic acid-inducible gene 1-like signalling, interleukins, interferon receptors and endogenous double strand RNA sensors. We propose that innate immune system activation starts in the olfactory bulb, is mediated by the retinoic acid-inducible gene 1-like signalling pathway in response to increased cytosolic double-strand RNA, leading to chemokines that recruit leukocytes to other brain regions to elicit an immune response. Our results fill in the gap between mitochondrial dysfunction and activation of the innate immune response, which has been reported by others. Our findings strongly suggest that immune system activation constitutes part of the Leigh syndrome pathomechanism, which is compatible with the improvement observed in mitochondrial disease patients following immune system-targeting interventions.

**Graphical Abstract:**

**Figure:**
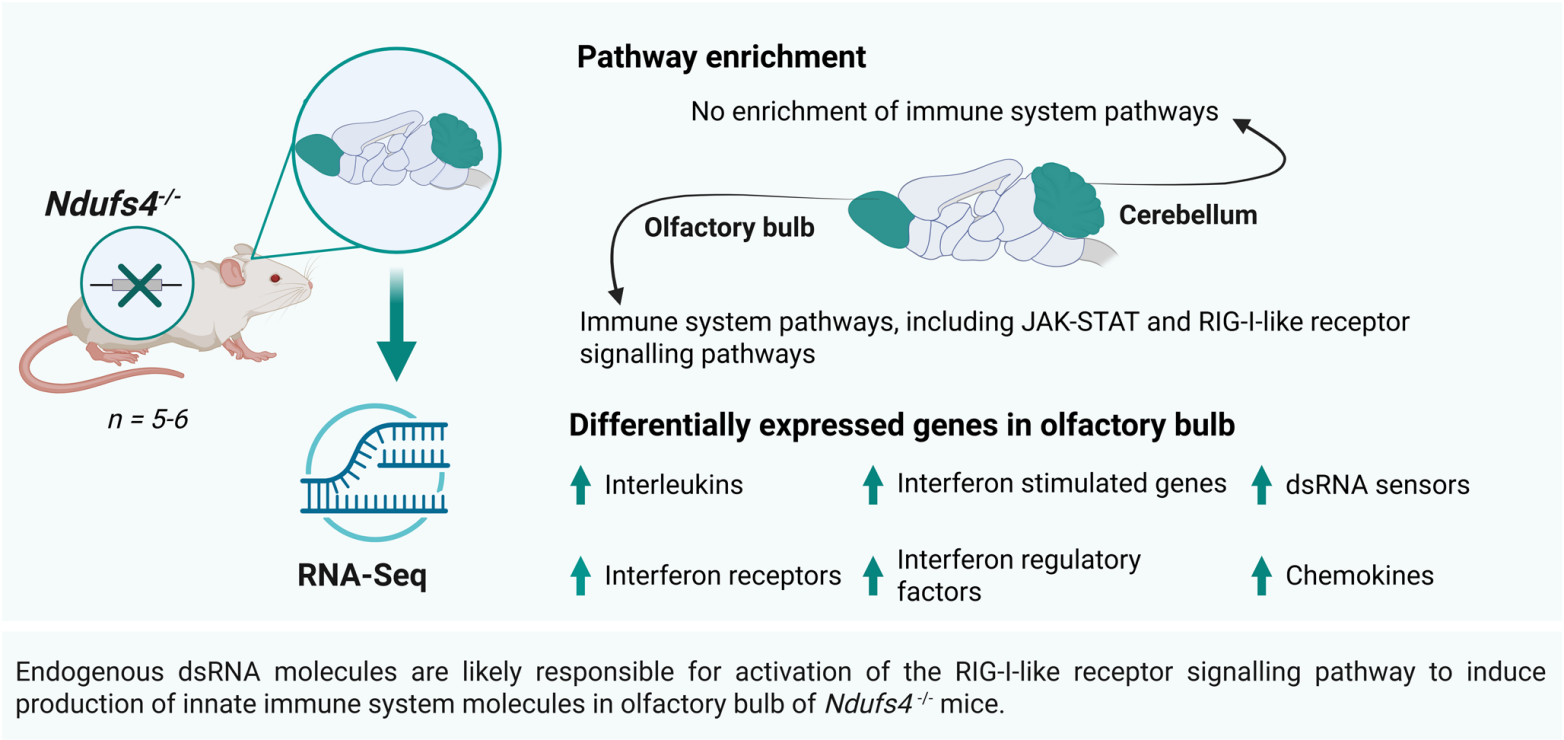
Enrichment of innate immune system pathways in olfactory bulb, but not cerebellum of *Ndufs4*^*−/−*^ mice. Transcriptomic analysis of *Ndufs4*^*−/−*^ mice brain tissues reveals enrichment of JAK-STAT and RIG-I-like receptor signalling pathways in olfactory bulb. We propose a pathomechanism of Leigh syndrome which starts with endogenous dsRNA molecules activating these signalling pathways, leading to chemokines that recruit leukocytes to other brain regions to elicit an immune response. Figure created with BioRender

**Supplementary Information:**

The online version contains supplementary material available at 10.1007/s10571-026-01681-2.

## Introduction

Mitochondrial diseases (MDs) represent the most common group of inborn errors of metabolism and often result from dysfunction of the oxidative phosphorylation (OXPHOS) system (Rahman and Rahman 2018). Patients with MD present heterogeneously, and even those with the same genetic mutations can present with different symptoms (Budde et al. 2003; Munaro et al. 1997; Lacbawan et al. 2000). The latter typically include failure to thrive, lactic acidosis, seizures, hypotonia, respiratory abnormalities and metabolic acidosis among many others (Distelmaier et al. 2009; Kapnick et al. 2018). Several MD symptoms involve the immune system, including recurring and severe infections (Tarasenko et al. 2017), low white blood cell counts, neutropenia and specific deficiencies in CD8^+^ T-cells and natural killer (NK) cells (Hanaford and Johnson 2022).

In human patients, infections have been reported as one of the environmental triggers for the onset of symptoms in MD (Suroviakova et al. 2025; Distelmaier et al. 2009), or to provoke worsening of symptoms in some cases of MD (Garone et al. 2011). In a cohort of 62 paediatric patients with probable and definite MD, 89% experienced recurrent or severe infections, including upper (47%) and lower (40%) respiratory tract infections, otitis media (24%) and, sinusitis (32%) (Tarasenko et al. 2017). Furthermore, a study investigating 221 children with MD highlighted sepsis (55%) and pneumonia (29%) as the most common causes of death (Eom et al. 2017). Several specific MDs have direct links to immune involvement, for example in POLG1 patients: chronic inflammation (Hanaford and Johnson 2022), and increased cerebrospinal fluid levels of interleukin (IL)−6, IL-8 and interferon-gamma (IFN-γ) (Hasselmann et al. 2010) have been reported, whereas neuroinflammation has been observed in cerebellum and brainstem in Friedreich ataxia patients (Khan et al. 2022). Moreover, inflammation-associated genes are upregulated in skeletal muscle from patients with thymidine kinase 2 deficiency (Kalko et al. 2014), whereas paediatric MD patients struggle to develop or maintain immunity (Kruk et al. 2019).

Paediatric Leigh syndrome (LS) is a relatively common MD (Lake et al. 2016). These patients present with a variety of symptoms including failure to thrive, lactic acidosis, metabolic acidosis, psychomotor retardation, and basal ganglia abnormalities, which ultimately lead to early death (Budde et al. 2003; Maruo et al. 2021; Leigh 1951). Recent evidence suggests that immune system aberrations are among the main drivers of LS pathology (Stokes et al. 2022; Hanaford et al. 2024). NADH: ubiquinone oxidoreductase subunit S4 (*Ndufs4*^*−/−*^*)* knockout (KO) mice are a widely used model to study LS pathomechanisms and intervention strategies (van de Wal et al. 2022, 2025; Yu et al. 2015; Hanaford et al. 2024; Adjobo-Hermans et al. 2020). The NDUFS4 protein constitutes an accessory subunit of mitochondrial complex I (CI) of the OXPHOS system, and plays a role in the assembly, regulation and/or stability of this complex (Kruse et al. 2008; Distelmaier et al. 2009; Scacco et al. 2003; Adjobo-Hermans et al. 2020; Calvaruso et al. 2012; Yin et al. 2024). KO mice present with symptoms similar to those of human CI deficiency, including encephalomyopathy, reduced body weight, hair-loss, retarded growth, blindness, cataracts, loss of motor skills and ataxia (Kruse et al. 2008).

Neuropathology is highly evident in KO mice, with brain tissue being affected at the proteome level (van de Wal et al. 2025), especially in lesion prone structures such as the olfactory bulb (OB), cerebellum and brainstem (Hanaford et al. 2024; Stokes et al. 2022; Quintana et al. 2012; Perry et al. 2021). Marked astrocytosis and microgliosis are also consistently observed in these three brain regions (Aguilar et al. 2022; Quintana et al. 2010). Furthermore, these brain regions are subject to molecular and metabolic changes, including a severe reduction in complex I activity and respiratory capacity (Kayser et al. 2016). Restoration of CI activity only in peripheral tissues through systemic AAV2/9-hNDUFS4, does not ameliorate the clinical phenotype of KO mice, whereas intracerebroventricular administration leads to a moderate improvement (Di Meo et al. 2017), which suggests that brain dysfunction is mainly responsible for the clinical phenotype in these animals. A substantial inflammatory response has also been observed in OB and cerebellum of KO mice (Ferrari et al. 2017; Hanaford et al. 2024), suggesting immune system involvement in this pathomechanism. In this context, previous studies proposed that CI serves as a key regulator of the innate immune system (Jin et al. 2014), and that retinal CI dysfunction increases innate immune and inflammatory markers, subsequently inducing the loss of retinal ganglion cell function and vision (Yu et al. 2015). Another study demonstrated that mice treated with the colony stimulating factor 1 receptor (CSF1R*)* inhibitor, pexidartinib, do not present with LS-like symptoms prior to death (Stokes et al. 2022), whereas the loss of IFN-γ modestly improved survival and delayed disease (Hanaford et al. 2024). Recent evidence suggests that LS pathology is not primarily driven by adaptive immune cells, but rather by the monocyte/macrophage innate immune system (Hanaford et al. 2025).

Here, we determined whether there are brain region-specific immune responses. To this end, we compared untargeted transcriptomics datasets obtained from the two most lesion prone brain regions (i.e. the OB and cerebellum) of *Ndufs4* KO and WT mice. Surprisingly, our data provide evidence that innate immunity-linked transcriptional responses are activated in the OB but not in the cerebellum. We propose a detailed pathomechanistic pathway underlying this activation.

## Materials and Methods

### Animal Model and Tissue Collection

Animal experiments were approved by the North-West University (NWU) Animal Research Ethics Committee (approval numbers NWU-00001–15-A5, NWU-00507-20-A5 and NWU-00788-23-A5). Heterozygous (*Ndufs4*^*+/−*^; B6.129S4-Ndufs4tm1.1Rpa/J) mice were acquired from The Jackson Laboratory (Bar Harbor, ME, USA) and used to breed wildtype (WT) and whole-body KO (*Ndufs4*^*−/−*^) males. All mice were bred and housed under controlled conditions at the specific pathogen-free unit of the vivarium of the Pre-Clinical Drug Development Platform (SAVC reg. no. FR15/13458) of the NWU. Mice were housed individually in ventilated cages at constant temperature (22 ± 1 °C), humidity (55 ± 10%) and light/dark cycles (12:12 h). Animals were fed a standard chow diet and had *ad libitum* access to water. Genotypes were confirmed with tail snips using PCR and the activity of complex I (Terburgh et al. 2021) was assessed using standard operating procedures based on existing methods (Rahman et al. 1996; Shepherd and Garland 1969; Janssen et al. 2007). To minimise sex-related variation only males were used in this study. Mice were euthanised between postnatal days (P) 45–51 via cervical dislocation after which tissues were collected and immediately snap-frozen in liquid nitrogen. Samples were stored at −80 °C until required for analysis.

### RNA Sequencing

Five mg tissue was used to isolate RNA with the MagMAX-96 Total RNA Isolation Kit (Cat. #AM1830; Thermo Fisher Scientific, Waltham, MA, USA) from OB and cerebellum. The quality was assessed using bleach agarose gel electrophoresis as described previously (Aranda et al. 2012). Briefly, a 1% (w/v) agarose gel was prepared in TAE buffer supplemented with 0.06% (v/v) commercial bleach and ethidium bromide (EtBr) at a final concentration of 5 µg/ml. Electrophoretic separation of RNA was carried out at 1 V/cm for 10 min, followed by 3 V/cm for 70 min. High quality RNA is indicated by the presence of two well-resolved bands corresponding to the 28 S rRNA (~ 4.7 kb) and the 18 S rRNA (~ 1.9 kb) with an approximate 28 S:18 S band intensity ratio of at least 2:1. Degraded or poor-quality RNA is suggested by smeared signal and/or a 28 S:18 S intensity ratio below 2:1. Due to technical limitations, samples across different tissues are not paired. RNA concentrations were quantified using the Qubit RNA HS Assay kit (Cat. #Q32852; Thermo Fisher Scientific) prior to reverse transcription into cDNA with the SuperScript IV VILO Master Mix kit (Cat. #11756050; Thermo Fisher Scientific) and following the manufacturer’s instructions. In brief, RNA samples were diluted to a final concentration of 3.3 ng/µL. Each 15 µL reaction contained 40 ng RNA and either 3 µL of SuperScript™ IV VILO™ Master Mix or SuperScript™ IV VILO™ No RT Master Mix. Thermal cycling conditions for reverse transcription were as follows (single cycle): primer annealing at 25 °C for 10 min, cDNA synthesis at 50 °C for 10 min, and enzyme inactivation at 85 °C for 5 min. The resulting cDNA was stored at − 20 °C until further use. RNA sequencing libraries and templates were generated using Ion Ampliseq Transcriptome Mouse Gene Expression Kit (Cat. #A36412; Thermo Fisher Scientific) and Ion Chef as previously described (Carino et al. 2019). In brief, cDNA was diluted to a final concentration of 0.8 ng/µL, and 15 µL of this dilution were used for construction of barcoded libraries. PCR amplification conditions were configured to 13 cycles with a 16-minute annealing/extension phase, in accordance with the manufacturer’s recommendations for pools comprising 6 145 − 24 576 primer pairs. During library construction, the Ion Chef™ normalized the concentration of the barcoded libraries to 100 pM (Carino et al. 2019). The resulting libraries were stored at 4 °C until use for template preparation. Template preparation was carried out using the Ion 540™ Kit – Chef in strict accordance with the manufacturer’s instructions. Briefly, libraries were diluted to a final concentration of 70 pM whereafter 25 µL were used per reaction. Ampliseq Mouse Transcriptome v1 was chosen as the reference library, and the target regions were specified as “Ampliseq Mouse Transcriptome v1 Designed”. Quality monitoring thresholds were configured as follows: bead loading (%) < 30; key signal (1–100) ≤ 30; useable sequence (%) ≤ 30. The number of flows was set to 500, as recommended by the manufacturer for 200‑base read sequencing. Loaded Ion 540 chips were sequenced using an Ion S5 Sequencer (Thermo Fisher Scientific). The first prepared chips were sequenced immediately or stored at 4 °C until the sequencing of the first chip was completed. Prior to its use, the second chip was allowed to equilibrate to room temperature for 20 min before sequencing was initiated.

Here, we focused on genes related to the immune system. The remaining transcriptome data will be presented in later manuscripts.

### Proteomic Analysis

The detailed methods for generating and processing the proteomics data have recently been described in detail (Khumalo et al. 2025). Briefly, a brain-region sample lysate was prepared, and proteins were reduced, alkylated and trypsin-digested. Next, peptides were analysed via liquid chromatography-tandem mass spectrometry (LC-MS/MS) using a data-independent acquisition (DIA) or Sequential Window Acquisition of all Theoretical Mass Spectra (SWATH) mode on a TripleTOF 5600 instrument (SCIEX, Framingham, MA, USA). Raw LC-MS/MS data was processed using Spectronaut (Version 17, Biognosys AG, Schlieren, Switzerland) with direct DIA against the Swiss-Prot mouse database. Identification was filtered to a 1% FDR, and label-free quantification was performed using crossrun normalisation. Data has been deposited in the ProteomeXchange Consortium via the PRIDE repository (https://www.ebi.ac.uk/pride/; with the dataset identifier PXD061439) (Perez-Riverol et al. 2022).

### Differential Gene Expression

Statistical and data analyses were performed using Microsoft Excel, custom R scripts (version 4.2.1) and DESeq2 (version 1.38.3) (Love et al. 2014).

In some cases, samples from the same tissue were analysed on different chips. To account for batch effects, chip number was controlled for by including it in the design argument of the DESeqDataSetFromMatrix function from DESeq2 (Love et al. 2014). Absolute reads below 10 were pre-filtered to remove rows with very few reads to increase the processing speed of count modelling within DESeq2. All default settings were used. Outliers were identified via PCA analyses using all detected transcripts per tissue. Samples outside the 95% confidence interval for a genotype were excluded. Differentially expressed genes (DEGs) were filtered using an adjusted *p*-value (*q*-value) ≤ 0.05 and FC ≤−2; ≥2; this yielded a list of significantly DEGs for each tissue.

After outlier exclusion, *n* = 6 per genotype and tissue, with the exception of WT cerebellum (*n* = 5).

### Pathway Analysis of DEGs

Significant DEGs were uploaded to WebGestalt (WEB-based GEne SeT AnaLysis Toolkit; version 2024; https://www.webgestalt.org/) to perform over-representation analysis (ORA) (Elizarraras et al. 2024). Kyoto encyclopedia of genes and genomes (KEGG) pathways were used as the functional database (https://www.kegg.jp/kegg/pathway.html). The reference list contained the 23,930 genes that were included in the sequencing panel. Parameters for the enrichment analysis were kept at the standard default settings: no redundancy removal was performed, and the minimum and maximum analytes for a category were kept at five and 2000, respectively. The Benjamini & -Hochberg (BH) procedure was used for multiple comparisons and a false discovery rate (FDR) of 0.05 was chosen for the significance level. The number of categories expected from set cover and the number of clusters (k) for k-medoid were set to 10.

### Differential Protein Abundance

Initial proteomics data processing was described previously (Khumalo et al. 2025). Here, we filtered our proteomics dataset on the basis of the lists of DEGs identified during the analyses described above. Differentially abundant proteins of statistical significance were defined by an adjusted (BH procedure) *p*-value ≤ 0.05 and/or FC ≤−2; ≥2.

Sample size was *n* = 9 (WT) and *n* = 8 (KO).

## Results

### Enriched Pathways for the OB are Involved in Innate Immune Responses

**Fig. 1:**
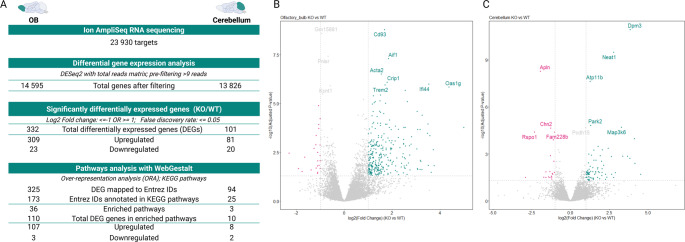
Total differentially expressed genes and pathways in OB and cerebellum after RNA-Seq. Differential gene expression analysis and pathway enrichment were performed on OB and cerebellum of KO and WT mice. **A** Using IonAmpliSeq RNA sequencing, the gene expression of 23 930 targets was determined. Genes with less than 10 reads were filtered to reduce the datasets to 14 595 and 13 826 for OB and cerebellum, respectively. Significant DEGs were determined by filtering for FC ≤−2 OR ≥ 2, and FDR ≤ 0.05, leaving a total of 332 and 101 significant DEGs for OB and cerebellum, respectively. Over-representation analysis was performed using WebGestalt and KEGG pathways. DEGs were first mapped to Entrez IDs before being annotated in KEGG pathways. In OB, the 36 enriched pathways included 110 DEGs, whereas 10 DEGs were mapped to the three enriched pathways of cerebellum. Volcano plots showing the log2 fold change (log2FC) of gene expression and the statistical significance of the differential expression analysis performed on WT and KO mice in (**B**) olfactory bulb and (**C**) cerebellum. The y-axis shows the − 10log of the adjusted p-values, while the x-axis shows the log2FC of genes. Each dot represents a gene, with blue (Log2FC ≥ 1) and pink (Log2FC ≤ −1) indicating genes with an adjusted p-value < 0.05. The top 10 significant genes are labelled. *DEGs* differentially expressed genes, *OB*  olfactory bulb, *KO * knockout, *WT* wildtype, *NS * non-significant (NS). Figure created with BioRender

Untargeted transcriptome analyses were performed on OB and cerebellum of WT and KO male mice. DEGs were identified, followed by pathway enrichment by means of ORA for both tissues. There were 332 and 101 DEGs in OB and cerebellum, respectively (Fig. 1). Differential expression of these genes was mostly upregulated. After conversion to Entrez IDs, 173 and 25 genes were annotated in the KEGG database for OB and cerebellum, respectively. For information on annotation of reference genes, see Supplementary Table 4, Additional File 1. In OB, 36 pathways were significantly enriched, most of which were related to viral infections and immune responses that comprised the IFN-mediated innate immune response (Table 1). Only three pathways were significantly enriched in cerebellum. The DEGs of the enriched pathways were mostly upregulated, except for three in OB and two in cerebellum, whose expression were downregulated.

**Table 1:** Enriched pathways in OB and cerebellum of KO mice

KEGG pathway	Gene set	Overlap	Enrich-ment ratio	FDR	Genes
OB					
Epstein-Barr virus infection (mmu05169)	201	23	4.84	7.74 × 10^− 8^	Cxcl10, Oas1g, Oas3, Oas1a, Irf7, Oas2, H2-Q7, Tap1, H2-Q4, H2-K1, Ddx58, H2-D1, Vim, Eif2ak2, H2-T24, Ccnd1, B2m, H2-M3, Cd40, Blnk, Ccnd2, Irf9, H2-T22
Herpes simplex infection (mmu05168)	185	20	4.57	1.70 × 10^− 6^	Oas1g, Oas3, Ccl5, Ifit1, Oas1a, Ccl12, Irf7, Oas2, H2-Q7, Tap1, H2-Q4, H2-K1, Ddx58, H2-D1, Eif2ak2, H2-T24, H2-M3, Sp100, Irf9, H2-T22
NOD-like receptor signaling pathway (mmu04621)	154	17	4.67	1.14 × 10^− 5^	Oas1g, Oas3, Ccl5, Ifi204, Oas1a, Ccl12, Irf7, Oas2, Gbp2, Gbp3, Gbp5, Gbp7, Naip2, Naip6, Cyba, Irf9, Gsdmd
Chemokine signaling pathway (mmu04062)	168	17	4.28	3.03 × 10^− 5^	Cxcl10, Ccl5, Cxcl11, Ccl12, Ccr7, Rac2, Cxcl16, Dock2, Cxcl5, Hck, Pik3r5, Gngt2, Cx3cr1, Ccl9, Cxcr4, Ncf1, Adcy4
Phagosome (mmu04145)	152	15	4.18	1.40 × 10^− 4^	Itga2, Fcgr4, H2-Q7, Tap1, H2-Q4, H2-K1, H2-D1, Fcgr1, Itgam, H2-T24, H2-M3, Cyba, Itga5, Ncf1, H2-T22
Influenza A (mmu05164)	152	15	4.18	1.40 × 10^− 4^	Cxcl10, Oas1g, Oas3, Ccl5, Oas1a, Ccl12, Irf7, Oas2, Rsad2, Ddx58, Eif2ak2, Mx2, Irf9, Trim25, Il33
Cytokine-cytokine receptor interaction (mmu04060)	262	20	3.23	1.46 × 10^− 4^	Cxcl10, Ccl5, Cxcl11, Ccl12, Ccr7, Eda2r, Csf2rb2, Csf2rb, Cxcl16, Il21r, Cxcl5, Cd40, Cx3cr1, Ccl9, Osmr, Il33, Cxcr4, Tgfb1, **Amh**,** Il20ra**
Viral myocarditis (mmu05416)	72	10	5.88	2.54 × 10^− 4^	H2-Q7, H2-Q4, Rac2, H2-K1, H2-D1, H2-T24, Ccnd1, H2-M3, Cd40, H2-T22
Antigen processing and presentation (mmu04612)	76	10	5.57	3.70 × 10^− 4^	H2-Q7, Tap1, H2-Q4, H2-K1, H2-D1, H2-T24, B2m, Psme2b, H2-M3, H2-T22
Human cytomegalovirus infection (mmu05163)	228	17	3.16	7.96 × 10^− 4^	Ccl5, Ccl12, H2-Q7, Tap1, H2-Q4, Rac2, H2-K1, H2-D1, H2-T24, Ccnd1, B2m, Gngt2, H2-M3, Cxcr4, Adcy4, H2-T22, Nfatc4
Allograft rejection (mmu05330)	53	8	6.39	8.63 × 10^− 4^	H2-Q7, H2-Q4, H2-K1, H2-D1, H2-T24, H2-M3, Cd40, H2-T22
Measles (mmu05162)	124	12	4.10	8.85 × 10^− 4^	Oas1g, Oas3, Oas1a, Irf7, Oas2, Ddx58, Eif2ak2, Ccnd1, Mx2, Ccnd2, Irf9, Msn
Kaposi sarcoma-associated herpesvirus infection (mmu05167)	189	15	3.36	9.12 × 10^− 4^	Irf7, H2-Q7, H2-Q4, H2-K1, H2-D1, Eif2ak2, H2-T24, Ccnd1, Hck, Pik3r5, Gngt2, H2-M3, Irf9, H2-T22, Nfatc4
Cell adhesion molecules (CAMs) (mmu04514)	155	13	3.55	1.61 × 10^− 3^	H2-Q7, Cd22, H2-Q4, H2-K1, Siglec1, H2-D1, Itgam, H2-T24, H2-M3, Cd40, Icam2, Pecam1, H2-T22
Human immunodeficiency virus 1 infection (mmu05170)	208	15	3.05	2.37 × 10^− 3^	Bst2, H2-Q7, Tap1, H2-Q4, Rac2, Trim30d, H2-K1, H2-D1, H2-T24, B2m, Gngt2, H2-M3, Cxcr4, H2-T22, Nfatc4
Autoimmune thyroid disease (mmu05320)	64	8	5.29	2.37 × 10^− 3^	H2-Q7, H2-Q4, H2-K1, H2-D1, H2-T24, H2-M3, Cd40, H2-T22
Cellular senescence (mmu04218)	164	13	3.35	2.37 × 10^− 3^	H2-Q7, H2-Q4, H2-K1, H2-D1, H2-T24, Ccnd1, H2-M3, Trpv4, Ccnd2, Rras, Tgfb1, H2-T22, Nfatc4
Natural killer cell mediated cytotoxicity (mmu04650)	104	10	4.07	2.86 × 10^− 3^	Fcgr4, Rac2, H2-K1, H2-D1, Fcer1g, Tyrobp, Hcst, Lcp2, Icam2, Ptpn6
Graft-versus-host disease (mmu05332)	55	7	5.39	4.75 × 10^− 3^	H2-Q7, H2-Q4, H2-K1, H2-D1, H2-T24, H2-M3, H2-T22
Osteoclast differentiation (mmu04380)	112	10	3.78	4.75 × 10^− 3^	Fcgr4, Trem2, Fcgr1, Tyrobp, Cyba, Blnk, Irf9, Lcp2, Ncf1, Tgfb1
Type I diabetes mellitus (mmu04940)	59	7	5.02	6.48 × 10^− 3^	H2-Q7, H2-Q4, H2-K1, H2-D1, H2-T24, H2-M3, H2-T22
Human T-cell leukemia virus 1 infection (mmu05166)	262	16	2.58	6.48 × 10^− 3^	H2-Q7, Tspo, H2-Q4, H2-K1, H2-D1, H2-T24, Ccnd1, H2-M3, Cd40, Ccnd2, Adcy4, Rras, Tgfb1, H2-T22, Nfatc4, **Bub1b**
Viral carcinogenesis (mmu05203)	189	13	2.91	6.91 × 10^− 3^	Irf7, H2-Q7, H2-Q4, H2-K1, H2-D1, Eif2ak2, H2-T24, Ccnd1, H2-M3, Sp100, Ccnd2, Irf9, H2-T22
Complement and coagulation cascades (mmu04610)	79	8	4.29	6.91 × 10^− 3^	Itgax, Serpinf2, Procr, Itgam, C1qb, Vwf, Pros1, Thbd
Leishmaniasis (mmu05140)	64	7	4.63	9.35 × 10^− 3^	Fcgr4, Fcgr1, Itgam, Cyba, Ncf1, Ptpn6, Tgfb1
Platelet activation (mmu04611)	112	9	3.40	1.57 × 10^− 2^	Itga2, Col3a1, Pik3r5, Pla2g4a, Fcer1g, Lcp2, Vwf, Adcy4, Apbb1ip
Cytosolic DNA-sensing pathway (mmu04623)	53	6	4.79	1.74 × 10^− 2^	Cxcl10, Ccl5, Irf7, Zbp1, Ddx58, Il33
Hepatitis C (mmu05160)	124	9	3.07	2.90 × 10^− 2^	Oas1g, Oas3, Ifit1, Oas1a, Irf7, Oas2, Ddx58, Eif2ak2, Irf9
Human papillomavirus infection (mmu05165)	339	17	2.12	2.90 × 10^− 2^	Oasl1, Oasl2, Itga2, H2-Q7, H2-Q4, H2-K1, H2-D1, Eif2ak2, H2-T24, Ccnd1, Mx2, H2-M3, Ccnd2, Irf9, Itga5, Vwf, H2-T22
Fc gamma R-mediated phagocytosis (mmu04666)	81	7	3.66	3.12 × 10^− 2^	Rac2, Fcgr1, Dock2, Hck, Pla2g4a, Ncf1, Arpc1b
Fluid shear stress and atherosclerosis (mmu05418)	127	9	3.00	3.14 × 10^− 2^	Ccl12, Rac2, Gstt2, Hmox1, Trpv4, Cyba, Pecam1, Ncf1, Thbd
JAK-STAT signaling pathway (mmu04630)	153	10	2.77	3.20 × 10^− 2^	Csf2rb2, Csf2rb, Ccnd1, Il21r, Ccnd2, Irf9, Osmr, Ptpn6, Gfap, **Il20ra**
Leukocyte transendothelial migration (mmu04670)	105	8	3.22	3.20 × 10^− 2^	Rac2, Itgam, Cyba, Pecam1, Msn, Cxcr4, Ncf1, Myl9
B cell receptor signaling pathway (mmu04662)	65	6	3.91	3.93 × 10^− 2^	Cd22, Cd72, Rac2, Pik3ap1, Blnk, Ptpn6
Hematopoietic cell lineage (mmu04640)	88	7	3.37	4.25 × 10^− 2^	Itga2, Cd22, Fcgr1, Itgam, Cd9, Itga5, Cd37
Neomycin, kanamycin and gentamicin biosynthesis (mmu00524)	5	2	16.93	4.72 × 10^− 2^	Hk3, Hk2
Cerebellum					
ECM-receptor interaction (mmu04512)	81	4	14.46	4.16 × 10^− 2^	Cd44, Col4a1, Col6a2, Thbs3
MAPK signaling pathway (mmu04010)	281	6	6.25	4.16 × 10^− 2^	**Fgf10**, Fgf3, Flna, Flnc, Map3k6, **Rasa2**
Focal adhesion (mmu04510)	190	5	7.71	4.16 × 10^− 2^	Col4a1, Col6a2, Flna, Flnc, Thbs3

The enriched pathways and genes that were differentially expressed in KO mice compared with WT controls were determined. All genes listed were upregulated in KO mice compared to WT controls, except those indicated in bold, which were downregulated. Overlap refers to the number of significant genes that are in the specific pathway. See Supplementary Table 5, Additional file 1 for more information on calculation of the enrichment ratio. *FDR* false discovery rate

### Interferon-Stimulated Genes (ISGs) and Chemokines are Upregulated in OB, but Not in Cerebellum

**Fig. 2:**
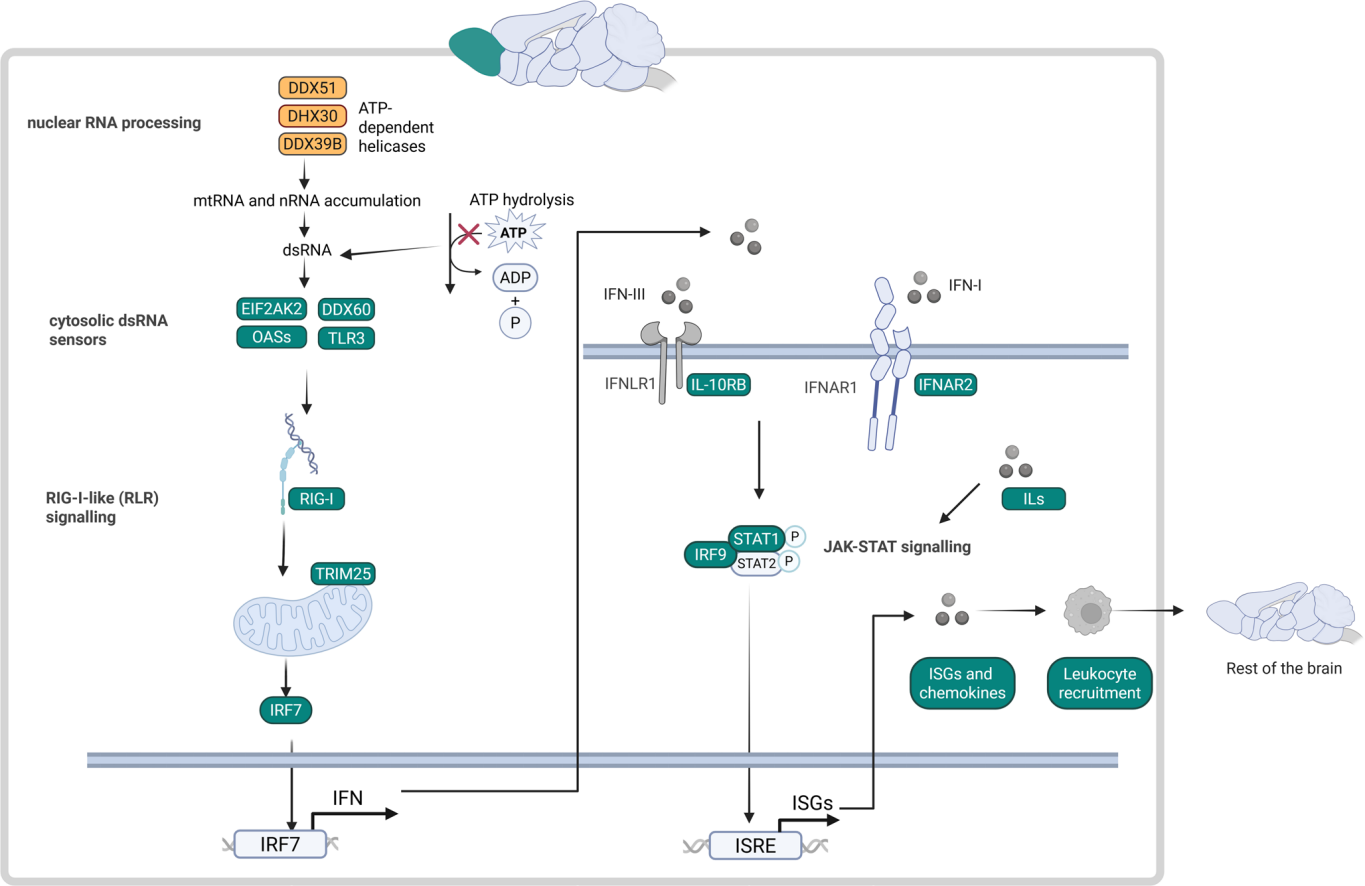
Differential expression of genes involved in the innate immune system in OB, but not in the cerebellum. To elucidate the mechanism underlying the enrichment of pathways associated with the innate immune system, we investigated the increased expression of a large subset of ISGs and chemokines in OB and worked our way upstream. This led us to propose a mechanism of activation of the innate immune system that starts with three ATP-dependent helicases involved in nuclear RNA processing, whose expression is decreased in OB. Four genes encoding cytosolic dsRNA sensors, as well as several genes encoding for proteins in the RIG-I-like signalling pathway were differentially expressed. Downstream of the RIG-I-like signalling pathway, genes encoding IL-10RB and IFNAR2, two subunits for interferon receptors were also differentially expressed in OB. In the JAK-STAT signalling pathway, we detected increased expression of *Irf9* and increased abundance of STAT1. The expression of 89 genes encoding ISGs, as well as genes encoding several chemokines was differential, with most being upregulated. Several genes indicating the presence of leukocytes and involved with leukocyte recruitment were also differentially expressed. Genes with increased differential expression are indicated in green, and those with decreased differential expression are indicated in yellow. *ISGs * interferon-stimulated genes, *IFN*  interferon, *IRF* interferon regulatory factor, *ISRE* interferon-sensitive response element. Figure created with BioRender

A large subset of interferon-stimulated genes (ISGs) was differentially expressed (see Supplementary Table 1, Additional File 1). A list of 445 ISGs (see Supplementary Table 3, Additional File 1) were compiled from literature (Dhir et al. 2018; West et al. 2015; Schoggins and Rice 2011; Keshavan et al. 2024; Yu et al. 2025), of which 89 were significantly differentially expressed in OB and one in cerebellum. In OB, fold changes ranged between − 1.67 and 32.02, with only five genes being downregulated. Among the 445 ISGs genes, 37 were also differentially abundant at the protein level in OB (see Supplementary Table 2, Additional File 1). Differential expression of 32 chemokines (see Supplementary Table 3, Additional File 1) was analysed, revealing that seven were significantly upregulated in the OB but not in the cerebellum. In light of the above, we focused on the OB in the remainder of the study. A stepwise elimination strategy was applied, starting from ISGs and chemokines and proceeding upstream to elucidate the mechanism involved. Where appropriate, we supplemented our gene expression data with proteomics data that were previously obtained (Khumalo et al. 2025). Through this approach, we identified the most likely pathomechanism presented by our data, which involved the activation of the innate immune system (Fig. 2):

Deletion of *Ndufs4* leads to the instability and reduced catalytic activity of CI (Kruse et al. 2008). This impairment of OXPHOS, the principal pathway for ATP generation, results in reduced cellular ATP production. We propose that the diminished ATP availability compromises the function of ATP-dependent enzymes, including ATP-dependent helicases. In the olfactory bulb (OB), three ATP-dependent helicases implicated in nuclear RNA processing exhibit reduced expression, a defect that may promote the accumulation of nuclear RNA (nRNA) and, consequently, double-stranded RNA (dsRNA). The increased dsRNA burden can, in turn, engage cytosolic dsRNA sensors and thereby initiate innate immune activation, starting with the retinoic acid-inducible gene 1 (RIG-I)-like signalling pathway. This in turn leads to interferon release and activation of the JAK-STAT signalling pathway; and finally, the resulting release of ISGs and chemokines results in leukocyte recruitment which can affect surrounding brain regions. Below, we present the results and discuss our discovery process, moving upstream from ISGs.

### Genes of the JAK-STAT Signalling Pathway are Upregulated in OB

ISGs can be directly stimulated by interferon regulatory factors (IRFs) (Schoggins and Rice 2011) and the JAK-STAT signalling pathway (Schneider et al. 2014). Therefore, we examined the expression of genes in these pathways. Four IRF genes, I*rf5*, I*rf7 Irf8 and Irf9*, presented with expression significantly increased, with fold changes ranging from 2.26 to 6.95 (see Supplementary Table 1, Additional File 1). *Stat1* was not included in our sequencing panel, but abundance of the STAT1 protein was increased in OB (*FC*: 2.45; *q*-value: 2.06 × 10^− 10^). *Stat2* expression was apparently increased (*FC*: 2.05), albeit not significantly. Other members of the JAK-STAT signalling pathway (i.e., *Tyk2*, *Jak1* and *Jak2*) were not differentially expressed.

The JAK-STAT signalling pathway is activated upon binding of IFNs to their receptors (Schneider et al. 2014). Therefore, we examined expression of these receptors in OB of KO mice next. This revealed that *Ifnar2* (*FC*: 1.62; *q*-value: 1.4 × 10^− 3^), *Ifngr2* (*FC*: 1.53; *q*-value: 3.84 × 10^− 2^) and *Il10rb* (*FC*: 1.62; *q*-value: 1.35 × 10^− 2^) were significantly upregulated. No IFN genes or proteins were detected in our datasets. However, two interferon regulators were detected: USP18 expression was markedly increased (*FC*: 8.99; *q*-value: 2.22 × 10⁻⁴), and ISG15 abundance was also significantly elevated (FC: 2.68; *q*-value: 1.02 × 10⁻⁷).

In addition to IFNs, the JAK-STAT signalling pathway can also be activated by ILs (Pazdrak et al. 1995; Sansone and Bromberg 2012). Several genes encoding IL receptors were differentially expressed in OB (see Supplementary Table 1, Additional File 1). All genes were upregulated, except for *Il20ra*, whose expression was downregulated. Protein levels of CNTFR were also slightly increased in OB (see Supplementary Table 2, Additional File 1).

### Genes Involved in the RIG-I Like Receptor Signalling Pathway are Upregulated in OB

Next, we determined whether genes upstream of the JAK-STAT signalling pathway were differentially expressed. We therefore investigated the expression of genes in three signalling pathways linked to the expression of IFNs: the toll-like receptor (TLR) signalling pathway (Kawai and Akira 2010), the cyclic GMP-AMP synthase-stimulator of interferon genes (cGAS-STING) (Chen et al. 2016), and the RIG-I-like receptor (RLR) signalling pathway (Yoneyama and Fujita 2009). Among the TLR genes, only *Tlr3* was differentially expressed (*FC*:1.91; *q*-value: 3.25 × 10^− 2^). None of the genes we investigated in the cGAS-STING pathway were differentially expressed (see Supplementary Table 3, Additional File 1 for list of genes in the cGAS-STING pathway that were investigated). However, several genes in the RLR pathway were differentially expressed (see Supplementary Table 1, Additional File 1). *Ddx58*, which encodes RIG-1, was upregulated in the OB of KO mice (*FC*: 3.11; *q*-value: 2.02 × 10^− 2^). Differential expression of *Ifih1*, encoding MDA5, as well as *Dhx58* trended upwards, but was not statistically significant. Gene expression of *Trim25* was increased in OB (*FC*: 2.25; *q*-value: 3.63 × 10^− 2^). *Irf7*, which is involved in several of the enriched pathways in these mice, was also upregulated in OB (*FC*: 6.95; *q*-value: 1.22 × 10^− 3^). The RLR pathway is typically activated in the presence of different RNA species (van Huizen and Gack 2025; Hornung et al. 2006; Jiang et al. 2011; Rehwinkel and Gack 2020; Yoneyama et al. 2004).

### Cytosolic dsRNA Sensors are Upregulated in OB

The genes encoding cytosolic dsRNA sensors, which detect dsRNA in the cytosol, were highly upregulated in OB of KO mice. *Eif2ak2*, which encodes protein kinase R (PKR), was upregulated (*FC*: 2.93; *q*-value: 3.26 × 10^− 2^). *Tlr3*, which specifically binds to dsRNA was also upregulated (as described earlier). Genes from seven 2’−5’ oligoadenylate synthetases (OASs), which also bind dsRNA, were highly upregulated with fold changes ranging from 4.02 to 20.77 (see Supplementary Table 1, Additional File 1). In addition, *Ddx60*, an RNA helicase, was upregulated in OB (*FC*: 4.85; *q*-value: 4.47 × 10^− 2^). DDX60 binds to dsRNA and complexes with RLRs to enhance signalling (Miyashita et al. 2011).

Next, we sought to identify possible sources of dsRNA, as these sources can trigger the RLR pathway.

### Genes Involved in Nuclear RNA Processing are Downregulated in OB

We identified several DEGs that are members of the DEAD box helicase family. These proteins are ATP-dependent RNA helicases and are involved in several points of nuclear RNA processing pathways (Srivastava et al. 2010; Booth et al. 2025; Bosco et al. 2021). In our dataset, the expression of *Dhx30* (*FC*: −1.26; *q*-value: 4.02 × 10^− 2^), *Ddx51* (*FC*: −1.44; *q*-value: 4.29 × 10^− 2^) and *Ddx39b* (*FC*: −1.25; *q*-value: 2.67 × 10^− 2^), was significantly downregulated. Protein abundance of DHX30 (*FC*: 1.88; *q*-value: 4.99 × 10^− 5^) and DDX39B (*FC*: −1.07; *q*-value: 2.69 × 10^− 2^) was also slightly but significantly altered.

### Unchanged Expression of tRNA Processing Factors and ADARs in OB

We investigated sources of tRNA fragments (tRFs) as potential triggers of the RLR pathway. We determined gene expression of genes encoding for proteins involved in tRNA processing, including angiogenin (*Ang)*, zinc phosphodiesterase ELAC protein 1 *(Elac1)*,* Elac2*, endoribonuclease dicer 1 *(Dicer1)* and 3-hydroxyacyl-CoA dehydrogenase type-2 *(Hsd17b10).* All genes were either not detected or not differentially expressed. ADAR enzymes are important for preventing MDA5, PKR and OASs from detecting endogenous dsRNA as nonself (Liddicoat et al. 2015; Matilainen et al. 2017; Chen and Hur 2022). None of the three adar genes (*Adar*, *Adarb1*, *Adarb2*) were differentially expressed in OB.

### Genes Indicating the Presence of Leukocytes

We then focused on genes indicating the presence of leukocytes. Expression of *Csf1r* was increased in OB of KO mice (*FC*: 1.72; *q*-value: 1.58 × 10^− 2^), whereas the expression of its ligands, *Csf1* (*FC*: 1.60) and *Il-34* (*FC*: 1.11) was also slightly but not significantly increased in OB. *Trem2* (*FC*: 3.28; *q*-value:1.13 × 10^− 6^) and *Tyrobp* (*FC*: 2.46; *q*-value: 1.91 × 10^− 4^), which are expressed mainly by microglia (a class of leukocytes) in the brain (Cobas-Carreno et al. 2025), were both significantly increased in OB. Accumulation of ionised calcium-binding adaptor molecule 1 (Iba1^+^) cells are commonly used to report on lesions in LS. Glial fibrillary acidic protein (GFAP) and translocator protein (TSPO) are also routinely used to report on astrogliosis and neuroinflammation (Milenkovic et al. 2018). Expression of *Aif1* which encodes Iba1^+^, *Gfap* and *Tspo* was significantly increased in OB (*FC*: 3.61, *q*-value: 5.07 × 10^− 8^, *FC*: 2.05; *q*-value: 1.45 × 10^− 2^ and *FC*: 3.72; *q*-value: 6.96 × 10^− 5^, respectively). Additionally, GFAP protein was significantly more abundant in OB (*FC*: 3.89; *q*-value: 1.54 × 10^− 6^).

Integrins are heterodimeric cell surface receptors that mediate cell adhesion (Hynes and Zhao 2000), including adhesion of leukocytes to endothelial cells during leukocyte migration. Several integrins and other proteins associated with leukocyte adhesion were upregulated in OB of KO mice (see Supplementary Table 1, Additional File 1).

## Discussion

Two highly affected brain regions (i.e. OB and cerebellum) of *Ndufs4* KO and WT mice were used to perform untargeted transcriptome analyses and pathway enrichment. Several pathways were enriched in OB, many of which are related to the innate immune response. Only three pathways, namely, MAPK signalling, ECM-receptor interaction and focal adhesion, were significantly enriched in cerebellum. Most of the DEGs in these pathways were upregulated, with noticeable overlap between enriched pathways. None of the genes involved in the innate immune response that we investigated in OB were differentially expressed in cerebellum, indicating that the innate immune response might be brain-region specific. This finding also suggests that the innate immune system is not the only driver of pathology and that other pathomechanisms might be involved in the cerebellum which is typically highly affected in MD (Hanaford et al. 2024; Ferrari et al. 2017; Stokes et al. 2022; Kayser et al. 2016).

### Interferons Activate the JAK-STAT Signalling Pathway to Produce ISGs in OB

Our data revealed the differential expression of numerous ISGs in OB of KO mice, with most being upregulated. ISGs are a set of genes critical to the antiviral defense system with a wide range of activities. They are activated by IFNs and interferon regulatory factors to reinforce IFN signalling (Wang et al. 2017; Au-Yeung and Horvath 2018; Orzalli et al. 2018; Rubio et al. 2013; Schoggins 2019; Schneider et al. 2014). ISG expression has been shown to be increased in whole blood of patients with MD in previous studies (Lepelley et al. 2021). Upregulation of ISGs in our KO mice, suggests that the OB upregulates immune pathways in response to mitochondrial disease. To understand the mechanism underlying the differential expression of ISGs in KO mice, we examined the expression of genes upstream of ISGs.

ISGs are induced upon activation of signalling pathways, including the JAK-STAT signalling pathway, when IFNs bind to their cell surface receptors (McNab et al. 2015). STAT1, STAT2 and IRF9 form the interferon-sensitive gene factor-3 (ISGF3) complex, which is a key player in JAK-STAT signalling (Stok et al. 2020; McNab et al. 2015; Schoggins 2019; Platanias 2005; Platanitis et al. 2019). Expression of *Irf9* and abundance of the STAT1 protein were increased in OB. Expression of *Stat2* was also slightly increased, although not significantly. These findings indicate that the JAK-STAT signalling pathway could be activated through binding of IFNs in KO mice.

The IFN-mediated innate immune response provides an initial protection against invading pathogens (Schneider et al. 2014; Alvarez et al. 2024; Riley and Tait 2020). There are three classes of IFNs (IFN-I to IFN-III). IFN-Is bind to interferon-α/β receptor (IFNAR), which consists of IFNAR1 and IFNAR2. IFN-IIs bind to the interferon-gamma receptor (IFNGR), which consists of the subunits IFNGR1 and IFNGR2, whereas IFN-IIIs bind to interferon-lambda receptor (IFNLR), which also consists of two subunits – IFNLR1 and IL-10Rβ (Mesev et al. 2019; Platanias 2005; Pestka et al. 1987, 2004). In our KO mice, expression of one gene from each of the three IFN receptors, including *Ifnar2*, *Ifngr2* and *Il10rb* was slightly upregulated. Several studies have reported that IFN-Is mediate the activation of the innate immune system in MD models (Hanaford et al. 2024; Lei et al. 2021) and patients (Warren et al. 2023). The upregulation of IFN receptors in our KO mice could indicate that more IFNs were available to bind to these receptors, even in the absence of increased IFN gene expression in our data. STAT–IRF9 complexes are able to sustain ISG expression even after interferon transcript levels have returned to baseline (Platanitis et al. 2019). Our data also indicated active negative regulation of type I interferon signalling: *Usp18* gene expression and ISG15 protein abundance was significantly elevated, both of which are key negative regulators of IFN-Is (Babadei et al. 2024). Together, these findings support a scenario in which interferons were transiently produced downstream of RIG I signalling, but effective feedback control normalized interferon transcript levels while leaving a strong ISG signature at the time of sampling.

We detected increased expression of five IL genes in OB of KO mice. Another group also reported increased expression of genes related to IL-1β and IL-1 production in MD patient peripheral blood mononuclear cells (Warren et al. 2023). The increase in expression of IL genes point to potentially increased release of ILs which could contribute to JAK-STAT signalling in addition to IFNs.

Overall, our data suggest that ISGs are stimulated directly through IRFs, or through the JAK-STAT signalling pathway, which is activated by either IFNs or ILs.

### The RIG-I-Like Receptor Pathway is the Likely Source of IFN Transcription

We next investigated which pattern recognition receptors (PRRs) could induce the transcription of IFNs. PRRs are nucleic acid sensors that recognise damage-associated molecular patterns (DAMPs), which are endogenous molecules such as DNA, RNA and proteins that can lead to a signalling cascade upon recognition by PRRs (Gong et al. 2020; Riley and Tait 2020). These signalling cascades in turn cause the release of cytokines, hormones and chemokines, including IFNs (Lee and Kim 2007; Li and Wu 2021). IFN-Is and IFN-IIIs are induced upon activation of the TLR and the RLR pathways (Lazear et al. 2015; Martinez-Espinoza and Guerrero-Plata 2023). IFN-I can additionally be produced upon activation of cyclic cGAS-STING. We therefore investigated the expression of TLR, cGAS-STING and RLR pathways.

Only one gene of the TLR pathway (*Tlr3*) and no genes of the cGAS-STING pathway were differentially expressed in OB of KO mice, providing weak to no evidence that these two pathways are the main routes of IFN transcription. However, the cGAS-STING pathway is regulated mainly post-translationally and cannot be completely excluded on the basis of our available data alone.

The RLR signalling pathway is responsible for detecting viral pathogens and generating innate immune responses, and consists of three members: RIG-1, melanoma differentiation-associated protein 5 (MDA5; also known as RLR2) and ATP-dependent RNA helicase DHX58 (also known as LGP2) (Rehwinkel and Gack 2020). We detected increased differential expression in OB of several genes in this pathway, including, *Ddx58*, which encodes RIG-1, *Trim25* and *Irf7*. Expression of *Ifih1*, encoding MDA5, and *Dhx58* tended to increase but was not statistically significant. Activation of RIG-1 or MDA5 leads to signalling of the mitochondrial antiviral signalling protein (MAVS) to recruit several signalling molecules, including IRF3/7 and NF-ĸB to induce transcription of IFN-Is and inflammatory cytokines (Chen and Hur 2022; Stok et al. 2020). Tripartite motif-containing 25 (TRIM25) is an E3 ligase proposed to ubiquitinate the RIG-I caspase activation and recruitment domains (CARDs) upon recognition of viral 5’-triphosphate-blunt-end dsRNA and may contribute to activation of MAVS (Alvarez et al. 2024; Stok et al. 2020). Similar to our findings, gene expression of the RLR signalling pathway has been reported to be increased in the retinas of MD patients (Warren et al. 2023) and *Ndufs4* KO mice (Yu et al. 2015).

Overall, these results indicate that the RLR pathway, but not the cGAS-STING or TLR pathways, is likely responsible for induction of transcription of type I and type III IFNs, especially since *Irf3* and *Irf7* are considered crucial IRFs required to induce transcription of IFN-Is (McNab et al. 2015).

### Potential Sources of dsRNA that Activate the RLR Pathway

Viral infections can trigger the immune response seen in KO mice as described above. However, the mice used in this study were housed in a pathogen-free area of the Pre-Clinical Drug Development Platform. In addition, WT and KO mice are housed together until sacrifice and sample collection, minimising the likelihood that only one genotype of mice was infected. Although the absence of an infection is only supported by our -omics data, we explored alternative triggers of RIG-I seen in OB tissue of KO mice.

RIG-I is activated by binding of dsRNA or 5’-triphosphate single strand RNA (ssRNA) (Sanchez David et al. 2019). Endogenous dsRNA can be explained by several dysregulated mechanisms, including epigenetic control, circular RNAs, RNA processing and degradation, and RNA pol III. Furthermore, changes to RNA modification, splicing inhibition and genotoxic stress could also contribute to dsRNA accummulation. Lastly, dsRNA can be released from mitochondria (Chen and Hur 2022).

### The dsRNA Sensors OASs, PKR, *Tlr3* and *Ddx60* were Upregulated in OB

A first indication of our hypothesis that dsRNA is the main source of immune system activation, lies with the family of OASs, and PKR. An effective immune response requires the synergistic action of several innate immune receptors, including RLRs, PKR and OASs (Ahmad et al. 2025). OAS members have been shown to be activated in response to different stressors by endogenous dsRNA in the absence of viral infection (Straub and Sampaio 2023). PKR is an IFN-inducible dsRNA sensor that can modulate RLR signalling (Chen and Hur 2022). Contact with inverted Alu repeats (IR-Alu) (Straub and Sampaio 2023) or mitochondrial double stranded RNA (mt-dsRNA) (Straub and Sampaio 2023) can activate PKR in uninfected cells.

Several cytosolic dsRNA sensors, including *Eif2ak2*, *Tlr3*,* Ddx60* and seven OASs were upregulated. DDX60 binds dsRNA and then forms complexes with RIG-I and MDA5 and enhances this signalling pathway, leading to IFN-β activation. It is specific to the RLR pathway and is not activated via the TICAM/TLR3 pathway (Miyashita et al. 2011). In mice, the OAS family consists of OAS1-3 and Oas-like proteins (OASL1 and OASL2) (Hornung et al. 2014). Upon detection of dsRNA, OASs are synthesised using ATP as a substrate, to activate RNaseL which in turn degrades cellular and viral RNA to ultimately inhibit protein synthesis and induce apoptosis (Hornung et al. 2014; Castelli et al. 1998). Since ATP is limited in KO mice, we expected oligoadenylate production and therefore also RNaseL activation to be decreased, but instead found OASs were highly upregulated, with *Oas1g and Oasl2* having fold changes of 20.77 and 11.39, respectively. *Oasl1* is known to inhibit the translation of IRF7 to negatively regulate antiviral immunity (Hornung et al. 2014). *Oasl1* and *Irf7* expression was increased in OB, but given that protein levels were not detected, translation of *Irf7* could still be inhibited in OB. OAS genes are also ISGs. The increased expression of OAS, PKR and TLR3 genes, points to dsRNA as the main source of endogenous RNAs that trigger the innate immune system activation. Therefore, we investigated endogenous sources of dsRNA and 5’-triphosphate ssRNAs that can trigger OASs, PKR and RIG-I.

### Incomplete Processing of Mitochondrial and Nuclear dsRNA as Triggers of RLRs

Expression of several genes of the DEAD box helicase family, namely *Dhx30*, *Ddx51* and *Ddx39b* were significantly decreased in the OB of KO mice, while the DHX30 protein was more abundant. The DEAD box proteins are ATP-dependent RNA helicases and involved in various steps of nuclear RNA (nRNA) processing (Srivastava et al. 2010; Bosco et al. 2021; Booth et al. 2025). DDX51 is involved in ribosomal maturation (Srivastava et al. 2010), while DDX39B aids in the process of mRNA export from of the nucleus (Clarke et al. 2025). Mutations in *Dhx30* and *Ddx39b* in humans have previously been reported to lead to neurodevelopmental disorders with symptoms that overlap with those of LS (Lessel et al. 2017; Booth et al. 2025). DHX30 is a mitoribosome assembly factor (Brischigliaro et al. 2024) that is involved in the processing of nRNAs, including those that code for mitoribosomal proteins (MRPLs and MRPSs). It is localised throughout the cytoplasm but is also found in mitochondria (Lessel et al. 2017; Wang and Bogenhagen 2006; Bosco et al. 2021). Depletion of *Dhx30* in cell lines leads to an increase of global translation of cytoplasmic ribosomes but a decrease in translation of mRNAs encoding for mitoribosomal proteins, as well as decreased expression and translation of mitochondrially encoded proteins (Bosco et al. 2021). Even in the absence of differential protein abundance, mitochondrial dysfunction as observed in *Ndufs4* KO mice could lead to aberrant RNA translation since DHX30 and other mitoribosomal proteins such as DDX28 contain redox sensitive cysteine residues (Brischigliaro et al. 2024; Kisty et al. 2023). Previous studies have shown that oxidation of cysteine residues on ribosomal proteins involved in transcription and translation processes, including RPLs and MRPLs, leads to a global, reversable decrease in cytoplasmic translation. This impact on translation is not due to changes in protein abundance but is rather due to a redox-sensitive regulatory mechanism (Topf et al. 2018). Furthermore, limited ATP might also directly impact the ability of a cell to recognise endogenous RNA. Although the exact mechanism is still unknown, ATPase activity by RIG-I is suggested to be involved in its ability to differentiate between endogenous and viral RNA (Lassig et al. 2015; Devarkar et al. 2018). It is proposed that mutations that decreases RIG-I’s ability to *bind* to ATP, results in defective signalling, while mutations that result in defective ATP *hydrolysis*, for example in Singleton-Merten Syndrome, result in binding to endogenous dsRNA and constitutive activation (Devarkar et al. 2018).

Taken together, limited ATP availability and increased reactive oxygen species (ROS) levels due to mitochondrial dysfunction could interfere with RNA helicase and other ribosomal protein function, leading to changes in RNA translation and possible accumulation of mtRNAs and nRNAs. These aberrant levels of dsRNA then bind to DDX60 and other RLR sensors to activate RIG-1 (Dhir et al. 2018; Miyashita et al. 2011). Additionally, decreased ATP hydrolysis could potentially interfere with endogenous RNA recognition, leading to activation of the RLR pathway by endogenous dsRNAs.

### The Pathway from Mitochondrial Dysfunction to Inflammation Markers in OB

Overall, our data suggest that the RLR pathway is activated in the OB of KO mice due to dsRNA accumulation from incomplete mitochondrial and nuclear dsRNA processing. Previous studies have primarily documented inflammation in *Ndufs4* KO mice, using whole brain analyses based on leukocyte presence and related markers (Stokes et al. 2022; Hanaford et al. 2023, 2025). We provide transcriptomic evidence supporting a mechanistic connection between mitochondrial dysfunction and inflammation markers. We did not observe this pathway in cerebellum. Since the OB is one of the highly affected brain regions in *Ndufs4* KO mice, and possibly one of the earliest regions to develop pathological symptoms, we hypothesise that this pathway is restricted to the OB (although we cannot exclude regions we did not investigate) and that its signal is diluted in whole brain analyses. Crucially, this initial OB response produces signalling molecules that could subsequently drive the whole brain inflammation reported by others (Martin-Perez et al. 2020; Johnson et al. 2013).

### Leukocyte Recruitment to OB Spreads Neuroinflammation

The expression of three genes encoding markers of neuroinflammation, *Aif1*,* Gfap* and *Tspo*, was increased in OB. Allograft inflammatory factor 1 (AIF1), also known as Iba1^+^, and GFAP are well-established markers of macrophages and astrocytes, respectively, whereas TSPO is a marker of activated microglia (Milenkovic et al. 2018). Immunohistochemistry against AIF1 and GFAP is commonly used in reports on KO mice and has been reported in OB, cerebellum, vestibular nuclei, motor cortex and whole brain (Felici et al. 2014; Johnson et al. 2013; Reynaud-Dulaurier et al. 2020; Martin-Perez et al. 2020), indicating that neuroinflammation is apparent in multiple brain regions. Although increased expression of *Aif1* and *Gfap* is believed to be due to increased activation, it could also indicate increased microglia and astroglia in the OB of KO mice.

*Trem2* and *Tyrobp* expression was increased in OB. Triggering receptor expressed on myeloid cells 2 (TREM2) is a transmembrane receptor expressed in microglia and astrocytes, and is critical for regulation of inflammatory processes (Cobas-Carreno et al. 2025). Upon activation of TREM2 and its adaptor protein TYRO protein tyrosine kinase-binding protein (TYROBP), several signalling proteins are activated, including the PI3K-Akt pathway which is involved in mTOR signalling (Cobas-Carreno et al. 2025). Ultimately, TREM2/TYROBP results in microglial activation (Cantoni et al. 2015).

We report increased expression of several chemokines in the OB of KO mice, alongside *Csf1r*, *Il-34* and *Csf1*. Chemokines induce directed leukocyte migration (Schall and Bacon 1994). Leukocyte proliferation has recently been implicated as a causal driver of LS pathogenesis; notably, leukocyte depletion via CSF1R inhibition in KO mice attenuated disease by reducing central nervous system lesions, suppressing seizures and extending survival (Stokes et al. 2022). CSF1R binds CSF1 and IL-34, which are essential for activation of innate immune cells and cytokine production. One of the enriched pathways in the OB was leukocyte trans endothelial migration (TEM), with all associated genes showing increased differential expression (see Supplementary Table 1, Additional File 1). Leukocyte TEM is crucial for innate immunity and inflammation (Schwartz et al. 2021) and involves a multi-step process in which leukocytes cross the endothelial barrier to reach infection sites (Gronloh et al. 2025). One of these steps involves the adhesion of leukocytes to endothelial cells, which is mediated by the interaction of integrins on both leukocytes and endothelial cells (Granger and Senchenkova 2010). Several genes encoding proteins involved in leukocyte adhesion including integrins, were upregulated in OB of KO mice, furthering our hypothesis of increased leukocyte TEM in OB (see Supplementary Table 1, Additional File 1).

Previous studies have revealed the involvement of multiple immune signalling pathways in *Ndufs4* KO pathogenesis (Jin et al. 2014; Martin-Perez et al. 2020; Stokes et al. 2022). However, recent evidence suggests that this pathology is driven mainly by the monocyte/macrophage innate immune system rather than T, B, or NK cells (Hanaford et al. 2025).

Building on the pathogenesis model proposed by Stokes et al. (2022), we suggest that elevated chemokine expression initiates leukocyte recruitment in the OB. Leukocytes subsequently disperse, activating other immune signalling pathways and releasing signalling molecules including macrophages, throughout the brain, ultimately contributing to the characteristic neurodegeneration and region-specific inflammation reported by others (Johnson et al. 2013, 2020; Martin-Perez et al. 2020; Aguilar et al. 2022; Hanaford et al. 2023, 2025; Stokes et al. 2022). Rapamycin’s documented effect of decreased leukocyte adhesion and migration in other disease models (Okamoto et al. 2016; Farkas et al. 2006; Nogueira de Francischi et al. 1993), may explain its therapeutic effects in KO mice by restricting leukocyte migration and thereby limiting propagation of immune activation, through the mechanism proposed in this study.

### Implications of this Study

These results expand on the understanding of the role of the immune system in MD, specifically the mechanism of pathology, supporting recent suggestions that the innate immune system rather than the adaptive immune response is a major driver of pathology (Hanaford et al. 2025). The use of KO mice at P50, which is near end-stage disease (Kruse et al. 2008), allowed us to investigate the mechanisms of evident pathology. This study clarifies why immune targeting interventions have shown substantial improvement in patients (Hanaford and Johnson 2022). Furthermore, it suggests several therapeutic targets upstream of the activated immune response. Understanding disease mechanisms is essential for the development or repurposing of drugs that slow innate immune signalling in response to endogenous dsRNA, for example, by targeting DAMP-sensing receptors. Additionally, DAMPs and PRRs could be explored as essential biomarkers for LS.

## Conclusion

Our study corroborates recent findings of the involvement of the innate immune system in MD. In our KO mouse model, we observed enrichment of immune-related pathways, and increased expression of ISGs and chemokines in OB, which aligns with proposals that upregulated type I ISGs are a key phenomenon in primary MD (Keshavan et al. 2024). We propose a mechanism for this activation: incomplete processing of mitochondrial/nuclear dsRNA, ROS sensitive regulation of ribosomal proteins, or reduced ATP hydrolysis might lead to accumulation of endogenous dsRNAs. These then activate the RLR signalling pathway to produce IFNs, which subsequently activate the JAK-STAT signalling pathway to induce ISG production. Some of these ISGs, functioning as chemokines, can recruit leukocytes to elicit an immune response in other brain regions. This clarifies the mechanisms of innate immune activation in sterile inflammation in LS, which is critical for novel treatment development.

This study made use of hypothesis generating methods (omics) to propose possible pathomechanisms that underly mitochondrial disease. These hypotheses provide starting points for follow-up, functional studies that further investigate the proposed pathomechanisms. Some of the limitations that follow-up studies are encouraged to address include this study’s reliance on gene expression data from only two brain regions, use of mostly one -omics platform and exclusively male mice. We report an unexpected difference in response to NDUFS4 KO mediated mitochondrial dysfunction in the OB compared to the cerebellum. The reason for this difference is not yet known, however, we proposed that the OB is the earliest brain region to be impacted by *Ndufs4* knockout and therefore might exhibit a different response than the cerebellum. However, we cannot exclude other possibilities such as technical limitations. For example, only a small section of tissue is used for RNA purification (5 mg). Since the OB is already a small structure, the entire structure of one hemisphere is used, while only a section of the entire cerebellum is used, resulting in less homogeneous samples for cerebellum. It is possible that this could increase inter-sample variance which would affect the significance statistics in cerebellum. Low-abundance immune related-proteins, such as cytokines, may have been masked in the proteomics data, and could explain why these proteins were not consistently detected in the proteomics data, although they were differentially expressed. Future work should address potential sex differences in immune enrichment, as reported in MD patients (Warren et al. 2023) and consider known transcriptional differences between human and mouse immune systems (Horton et al. 2023). Future research should also involve quantifying systemic IFN protein levels, exploring other tissues and validating these findings across varying MD models and patients, ideally including time course-analysis to track immune response evolution.

## Supplementary Information

Below is the link to the electronic supplementary material.

Supplementary Material 1

## Data Availability

The datasets used and/or analysed during the current study are available from the corresponding author on reasonable request.

## Abbreviations

OASs: 2’-5’ oligoadenylate synthetases
AIF1: Allograft inflammatory factor 1
BH: Benjamini & Hochberg
CARDs: Caspase activation and recruitment domains
csf1r: Colony stimulating factor 1 receptor
CI: Complex I
cGAS-STING: Cyclic GMP-AMP synthase-stimulator of interferon genes
DAMPs: Damage-associated molecular patters
DIA: Data-independent acquisition
DEGs: Differentially expressed genes
FDR: False discovery rate
GFAP: Glial fibrillary acidic protein
ISREs: IFN-stimulated response elements
IRFs: Interferon regulatory factors
ISGs: Interferon stimulated genes
IFN‑γ: Interferon-gamma
IFNLR: Interferon-lambda receptor
ISGF3: Interferon-sensitive gene factor-3
IFNAR: Interferon-α/β receptor
IL: Interleukin
Iba1+: Ionized calcium-binding adaptor molecule 1
KO: Knockout
LS: Leigh syndrome
LC-MS/MS: Liquid chromatography-tandem mass spectrometry
MDA5: Melanoma differentiation-associated protein 5
MAVS: Mitochondrial antiviral signalling protein
MD: Mitochondrial disease
mt-dsRNA: Mitochondrial double stranded RNA
NDUFS4: NADH: Ubiquinone oxidoreductase subunit S4
NK: Natural killer
nRNA: Nuclear RNA
OB: Olfactory bulb
ORA: Over-representation analysis
OXPHOS: Oxidative phosphorylation
PRRs: Pattern recognition receptors
PKR: Protein kinase R
ROS: Reactive oxygen species
RIG-I: Retinoic acid-inducible gene 1
RLR: RIG-I-like receptor
SWATH: Sequential window acquisition of all theoretical mass spectra
ssRNA: single strand RNA
IFNGR: The interferon‑gamma receptor
TLR: Toll-like receptor
TEM: Transendothelial migration
Tspo: Translocator protein
TREM2: Triggering receptor expressed on myeloid cells 2
TRIM25: Tripartite motif-containing 25
tRFs: tRNA fragments
TYROBP: TYRO protein tyrosine kinase binding protein
WT: Wildtype
