# Supplementary material for: Leigh Syndrome Pathomechanism Involves Region-Specific Innate Immune Activation in Ndufs4 Knockout Mice
Source: Cell Mol Neurobiol. 2026 Feb 4;46:42. doi: 10.1007/s10571-026-01681-2 (PMC12917085; doi:10.1007/s10571-026-01681-2)
Supplement: Supplementary file 1 — Supplementary Material 1 [file 10571_2026_1681_MOESM1_ESM.pdf]

Supplementary information

Leigh syndrome pathomechanism involves region-specific innate immune activation in Ndufs4 knockout mice

Cellular and Molecular Neurobiology

Belinda R. Fouché, Sibonelo G. Khumalo, Werner J.H. Koopman, and Marianne Venter

Correspondence to: Marianne Venter

Marianne.pretorius@nwu.ac.za

Biomedical and Molecular Metabolism Research (BioMMet), North-West University, Potchefstroom, South Africa.

Supplementary Table 1 Genes involved in innate immune system pathways in OB of KO mice

Gene symbol	Gene name	Transcriptomics	
		Fold change	Adjusted p-value
**ISGs**			
Cxcl10	C-X-C motif chemokine ligand 10	32.08	1.69×10^{-4}
Oas1g	2'-5' oligoadenylate synthetase 1G	20.77	1.46×10^{-6}
Oas3	2'-5' oligoadenylate synthetase 3	13.29	2.36×10^{-3}
Ccl5	C-C motif chemokine ligand 5	12.45	3.40×10^{-2}
Ifi44	Interferon-induced protein 44	11.63	1.02×10^{-6}
Cxcl11	Chemokine (C-X-C motif) ligand 11	11.42	1.48×10^{-2}
Oasl2	2'-5' oligoadenylate synthetase-like 2	11.39	1.13×10^{-4}
Ifit1	Interferon-induced protein with tetratricopeptide repeats 1	11.17	3.42×10^{-4}
Mx1	MX dynamin-like gtpase 1	9.00	2.36×10^{-2}
Usp18	Ubiquitin specific peptidase 18	8.99	2.20×10^{-4}
Ifi204	Interferon activated gene 204	8.36	2.16×10^{-2}
Rtp4	Receptor transporter protein 4	7.04	5.09×10^{-4}
Irf7	Interferon regulatory factor 7	6.95	1.22×10^{-3}
Itga2	Integrin alpha 2	6.72	2.54×10^{-2}
Oas2	2'-5' oligoadenylate synthetase 2	6.23	8.21×10^{-6}
Pyhin1	Pyrin and HIN domain-containing protein 1	6.10	1.44×10^{-2}
Nlrc5	NLR family, CARD domain containing 5	5.88	1.69×10^{-4}
Gbp2	Guanylate binding protein 2	5.86	2.17×10^{-6}
Zbp1	Z-DNA binding protein 1	5.69	1.58×10^{-2}
Gbp3	Guanylate binding protein 3	5.63	1.71×10^{-4}
Bst2	Bone marrow stromal cell antigen 2	5.61	1.09×10^{-3}
Rsad2	Radical S-adenosyl methionine domain containing 2	5.60	1.23×10^{-3}

Gene symbol	Gene name	Transcriptomics	
		Fold change	Adjusted p-value
Gbp5	Guanylate binding protein 5	5.51	3.78 x10^{-3}
Ddx60	Dexd/H box helicase 60	4.85	4.47 x10^{-2}
Trim30a	Tripartite motif-containing 30A	4.43	1.43 x10^{-4}
Tap1	Transporter 1, ATP-binding cassette, sub-family B (MDR/TAP)	4.15	9.38 x10^{-3}
Xaf1	XIAP associated factor 1	4.00	8.01 x10^{-4}
Psmb9	Proteasome (prosome, macropain) subunit, beta type 9 (large multifunctional peptidase 2)	4.00	1.33 x10^{-2}
Gbp4	Guanylate binding protein 4	3.91	1.03 x10^{-3}
Ifi203	Interferon activated gene 203	3.89	2.38 x10^{-3}
Procr	Protein C receptor, endothelial	3.87	1.91 x10^{-3}
Rnf213	Ring finger protein 213	3.85	7.93 x10^{-3}
Parp14	Poly (ADP-ribose) polymerase family, member 14	3.83	2.08 x10^{-3}
Psmb8	Proteasome (prosome, macropain) subunit, beta type 8 (large multifunctional peptidase 7)	3.71	1.69 x10^{-4}
Csf2rb	Colony stimulating factor 2 receptor, beta, low-affinity (granulocyte-macrophage)	3.56	3.79 x10^{-2}
Epsti1	Epithelial stromal interaction 1	3.54	5.78 x10^{-3}
Parp9	Poly (ADP-ribose) polymerase family, member 9	3.36	9.40 x10^{-4}
Lgals9	Lectin, galactose bindiuing, soluble 9	3.14	3.28 x10^{-5}
Ddx58		3.11	2.02 x10^{-2}
Lgals3bp	Lectin, galactoside-binding, soluble, 3 binding protein	3.07	2.42 x10^{-2}
Trim21	Tripartite motif-containing 21	2.96	1.66 x10^{-2}
Eif2ak2	Eukaryotic translation initiation factor 2-alpha kinase 2	2.93	3.26 x10^{-2}
Slfn5	Schlafen 5	2.93	5.64 x10^{-4}
Dtx3l	Deltex 3-like, E3 ubiquitin ligase	2.88	1.31 x10^{-2}
Gbp7	Guanylate binding protein 7	2.80	4.09 x10^{-2}
Fgl2	Fibrinogen-like protein 2	2.79	4.27 x10^{-3}
Irgm1	Immunity-related gtpase family M member 1	2.74	5.99 x10^{-3}
Irf8	Interferon regulatory factor 8	2.74	1.54 x10^{-3}
B2m	Beta-2 microglobulin	2.65	4.27 x10^{-2}
Slc15a3	Solute carrier family 15, member 3	2.64	2.39 x10^{-2}
Pla2g4a	Phospholipase A2, group IVA (cytosolic, calcium-dependent)	2.64	1.02 x10^{-4}
P2ry6	Pyrimidinergic receptor P2Y, G-protein coupled, 6	2.57	2.99 x10^{-2}
Pik3ap1	Phosphoinositide-3-kinase adaptor protein 1	2.55	2.02 x10^{-2}
Mx2	MX dynamin-like gtpase 2	2.52	4.52 x10^{-2}
Zc3hav1	Zinc finger CCCH type, antiviral 1	2.49	2.23 x10^{-3}
Ube2l6	Ubiquitin-conjugating enzyme E2L 6	2.48	1.40 x10^{-3}
Cd40	CD40 antigen	2.46	4.10 x10^{-2}
Sp100	Nuclear antigen Sp100	2.46	1.28 x10^{-2}
Irf9	Interferon regulatory factor 9	2.33	9.65 x10^{-3}
Irf5	Interferon regulatory factor 5	2.26	1.75 x10^{-2}
Lcp2	Lymphocyte cytosolic protein 2	2.25	2.02 x10^{-2}
Trim25	Tripartite motif-containing 25	2.25	3.63 x10^{-2}
Ifi27	Interferon, alpha-inducible protein 27	2.14	2.62 x10^{-3}

Gene symbol	Gene name	Transcriptomics	
		Fold change	Adjusted p-value
Tnfaip2	Tumor necrosis factor, alpha-induced protein 2	2.12	3.79×10^{-2}
Ptpn6	Protein tyrosine phosphatase, non-receptor type 6	2.10	2.61×10^{-2}
Hk2	Hexokinase 2	2.05	4.30×10^{-3}
Ifi35	Interferon-induced protein 35	2.01	2.68×10^{-2}
Tor3a	Torsin family 3, member A	1.97	4.34×10^{-3}
Psmb10	Proteasome (prosome, macropain) subunit, beta type 10	1.95	8.01×10^{-4}
Parp12	Poly (ADP-ribose) polymerase family, member 12	1.90	9.59×10^{-3}
Cfh	Complement component factor h	1.89	2.54×10^{-2}
Rhoc	Ras homolog family member C	1.87	7.44×10^{-3}
Tmem176a	Transmembrane protein 176A	1.83	3.42×10^{-2}
Tapbp	TAP binding protein	1.78	1.22×10^{-3}
Ly6e	Lymphocyte antigen 6 family member E	1.74	4.40×10^{-3}
Vcam1	Vascular cell adhesion molecule 1	1.65	4.60×10^{-2}
Ifnar2	Interferon (alpha and beta) receptor 2	1.62	1.40×10^{-3}
Zfp36l2	Zinc finger protein 36, C3H type-like 2	1.60	3.21×10^{-2}
Vamp8	Vesicle-associated membrane protein 8	1.55	2.84×10^{-2}
Golm1	Golgi membrane protein 1	1.48	6.52×10^{-3}
Cnp	2',3'-cyclic nucleotide 3' phosphodiesterase	1.46	3.58×10^{-3}
Tdrd7	Tudor domain containing 7	1.44	1.58×10^{-2}
Lap3	Leucine aminopeptidase 3	1.41	1.28×10^{-2}
Sdcbp	Syndecan binding protein	1.35	5.76×10^{-3}
Rapgef6	Rap guanine nucleotide exchange factor (GEF) 6	-1.32	4.81×10^{-2}
Fubp1	Far upstream element (FUSE) binding protein 1	-1.35	2.31×10^{-2}
Atp10a	Atpase, class V, type 10A	-1.49	3.78×10^{-3}
Cdkn1a	Cyclin dependent kinase inhibitor 1A	-1.51	4.06×10^{-2}
Auts2	Autism susceptibility candidate 2	-1.67	2.54×10^{-2}
**Chemokines**			
*Ccl5**	C-C motif chemokine ligand 5	12.45	3.40×10^{-2}
Ccl9	C-C motif chemokine ligand 9	2.31	2.49×10^{-2}
Ccl12	C-C motif chemokine ligand 12	8.24	2.15×10^{-3}
Cxcl5	C-X-C motif chemokine ligand 5	2.70	4.58×10^{-2}
*Cxcl10**	C-X-C motif chemokine ligand 10	32.08	1.69×10^{-4}
Cxcl11	C-X-C motif chemokine ligand 11	11.42	1.48×10^{-2}
Cxcl12	C-X-C motif chemokine ligand 12	1.82	1.93×10^{-4}
**Interferon-regulatory factors (IRFs)**			
Irf1	Interferon regulatory factor 1	1.54	NS
Irf2	Interferon regulatory factor 2	NS	NS
Irf3	Interferon regulatory factor 3	NS	NS
Irf4	Interferon regulatory factor 4	NS	NS
Irf5	Interferon regulatory factor 5	2.26	1.75×10^{-2}
Irf6	Interferon regulatory factor 6	2.79	NS
Irf7	Interferon regulatory factor 7	6.95	1.22×10^{-3}

Gene symbol	Gene name	Transcriptomics	
		Fold change	Adjusted p-value
Irf8	Interferon regulatory factor 8	2.74	1.54×10^{-3}
Irf9	Interferon regulatory factor 9	2.33	9.65×10^{-3}
**JAK-STAT signalling pathway**			
Stat1	Signal transducer and activator of transcription 1	ND	ND
Stat2	Signal transducer and activator of transcription 2	2.04	NS
Jak1	Tyrosine-protein kinase JAK1	NS	NS
Jak2	Tyrosine-protein kinase JAK2	NS	NS
Tyk2	Non-receptor tyrosine-protein kinase TYK2	NS	NS
**Interferon receptors**			
Ifnar1	Interferon alpha/beta receptor 1	NS	NS
Ifnar2	Interferon alpha/beta receptor 2	1.62	1.40×10^{-3}
Ifngr1	Interferon gamma receptor 1	NS	NS
Ifngr2	Interferon gamma receptor 2	1.53	3.84×10^{-2}
Ifnlr1	Interferon lambda receptor 1	1.57	NS
Il10rb	Interleukin-10 receptor subunit beta	1.62	1.35×10^{-2}
**Interleukins**			
Csf2rb2	Colony stimulating factor 2 receptor, beta 2, low-affinity (granulocyte-macrophage)	4.02	1.08×10^{-2}
Csf2rb	Colony stimulating factor 2 receptor, beta, low-affinity (granulocyte-macrophage)	3.56	3.79×10^{-2}
Il21r	Interleukin 21 receptor	2.70	2.77×10^{-2}
Osmr	Oncostatin M receptor	2.25	3.70×10^{-3}
Il10rb	Interleukin 10 receptor, beta	1.62	1.35×10^{-2}
Cntfr	Ciliary neurotrophic factor receptor	NS	NS
Il20ra	Interleukin 20 receptor, alpha	-2.17	1.59×10^{-2}
**Toll-like receptors**			
Tlr1	Toll like receptor 1	2.62	NS
Tlr2	Toll like receptor 2	2.14	NS
Tlr3	Toll like receptor 3	1.91	3.25×10^{-2}
Tlr4	Toll like receptor 4	NS	NS
Tlr5	Toll like receptor 5	1.53	NS
Tlr6	Toll like receptor 6	NS	NS
Tlr7	Toll like receptor 7	NS	NS
Tlr8	Toll like receptor 8	2.77	NS
Tlr9	Toll like receptor 9	2.26	NS
Tlr10	Toll like receptor 10	ND	ND
Tlr11	Toll like receptor 11	NS	NS
Tlr12	Toll like receptor 12	2.05	NS
Tlr13	Toll like receptor 13	1.63	NS
**RIG-I like receptor signalling pathway**			
Ddx58	RNA sensor RIG-I	3.11	2.02×10^{-2}
Ifih1	Interferon induced with helicase C domain 1	2.72	NS
Dhx58	Dexh-box helicase 58	1.51	NS

Gene symbol	Gene name	Transcriptomics	
		Fold change	Adjusted p-value
Trim25	Tripartite motif-containing 25	2.25	3.63×10^{-2}
Irf7	Interferon regulatory factor 7	6.95	1.22×10^{-3}
Nfkb2	Nuclear factor of kappa light polypeptide gene enhancer in B cells 2	1.62	3.43×10^{-2}
**Endogenous dsRNA sensors**			
Eif2ak2	Eukaryotic translation initiation factor 2-alpha kinase 2	2.93	3.26×10^{-2}
Oas1a	2'-5'-oligoadenylate synthase 1A	8.29	2.55×10^{-5}
Oas1b	2'-5' oligoadenylate synthetase 1B	4.02	5.32×10^{-2}
Oas1c	Inactive 2'-5' oligoadenylate synthetase 1C	NS	NS
Oas1d	Inactive 2'-5'-oligoadenylate synthase 1D	NS	NS
Oas1e	2'-5'olygoadenylate synthetase 1E, 42kd isoform	NS	NS
Oas1f	2'-5' oligoadenylate synthetase 1F	NS	NS
Oas1g	2'-5' oligoadenylate synthase 1G	20.77	1.46×10^{-6}
Oas1h	2'-5' oligoadenylate synthetase 1H	NS	NS
Oas2	2'-5'-oligoadenylate synthase 2	6.23	8.21×10^{-6}
Oas3	2'-5'-oligoadenylate synthase 3	13.29	2.36×10^{-3}
Oasl1	2'-5'-oligoadenylate synthase-like protein 1	12.02	4.42×10^{-4}
Oasl2	2'-5'-oligoadenylate synthase-like protein 2	11.39	1.13×10^{-4}
Ddx60	Dexd/H box helicase 60	4.85	4.47×10^{-2}
**RNA processing**			
Dhx30	DExH-box helicase 30	-1.26	4.02×10^{-2}
Ddx51	DEAD box helicase 51	-1.44	4.29×10^{-2}
Ddx39b	DEAD box helicase 39b	-1.25	2.67×10^{-2}
**Leukocytes (transendothelial migration, adhesion and proliferation) and integrins**			
Rac2	Rac family small gtpase 2	3.58	7.93×10^{-3}
Itgam	Integrin alpha M	2.87	2.80×10^{-5}
Cyba	Cytochrome b-245, alpha polypeptide	2.37	1.49×10^{-3}
Pecam1	Platelet/endothelial cell adhesion molecule 1	2.17	2.15×10^{-3}
Msn	Moesin	2.15	6.69×10^{-3}
Cxcr4	C-X-C motif chemokine receptor 4	2.11	2.45×10^{-2}
Ncf1	Neutrophil cytosolic factor 1	2.10	1.33×10^{-2}
Myl9	Myosin, light polypeptide 9, regulatory	2.08	2.07×10^{-4}
Csf1r	colony stimulating factor 1 receptor	1.72	1.58×10^{-2}
Csf1	colony stimulating factor 1 (macrophage)	1.60	NS
Il34	interleukin 34	1.11	NS
Aif1	allograft inflammatory factor 1	3.61	5.07×10^{-8}
Gfap	glial fibrillary acidic protein	2.05	1.45×10^{-2}
Itgax	integrin alpha X	9.62	4.01×10^{-5}
Cd44	CD44 antigen	3.10	NS
Itgam	integrin alpha M	2.87	2.80×10^{-5}
Itgal	integrin alpha L	2.42	NS
Sele	selectin, endothelial cell	2.24	NS
Icam2	intercellular adhesion molecule 2	2.21	3.79×10^{-2}

Gene symbol	Gene name	Transcriptomics	
		Fold change	Adjusted p-value
Pecam1	platelet/endothelial cell adhesion molecule 1	2.17	2.15×10^{-3}
Itgb2	integrin beta 2	1.88	NS
Vcam1	vascular cell adhesion molecule 1	1.65	4.60×10^{-2}
Trem2	Triggering receptor expressed on myeloid cells 2	3.28	1.13×10^{-6}
Tyrobp	TYRO protein tyrosine kinase binding protein	2.46	1.91×10^{-4}
Tspo	Translocator protein	3.72	6.96×10^{-5}

Transcriptomics data on the differential expression of genes in the pathways leading to transcription of ISGs and chemokines. *chemokines that are also ISGs. Adjusted p-value <0.05. NS = not significant; ND = not determined

Supplementary Table 2 Proteins that are involved in innate immune system pathways in OB of KO mice

Gene symbol	Gene name	Proteomics	
		Fold change	q-value
**Interferon stimulated genes**			
CDKN1B	Cyclin dependent kinase inhibitor 1b	-1.53	2.11 x10^{-2}
BAX	Bcl2-associated x protein	-1.45	8.16 x10^{-4}
CLIC4	Chloride intracellular channel 4	-1.44	4.23 x10^{-4}
SEPTIN4		-1.36	5.56 x10^{-4}
FUBP1	Far upstream element (fuse) binding protein 1	-1.35	2.22 x10^{-4}
MAP1B	Microtubule-associated protein 1b	-1.34	2.03 x10^{-9}
CD47	Cd47 antigen (rh-related antigen, integrin-associated signal transducer)	-1.29	7.63 x10^{-4}
ISOC1	Isochorismatase domain containing 1	-1.21	7.06 x10^{-4}
PDXK	Pyridoxal (pyridoxine, vitamin b6) kinase	-1.21	4.45 x10^{-6}
PPA1	Pyrophosphatase (inorganic) 1	-1.19	2.34 x10^{-3}
PPP5C	Protein phosphatase 5, catalytic subunit	-1.17	2.16 x10^{-2}
PSMB2	Proteasome (prosome, macropain) subunit, beta type 2	-1.14	4.16 x10^{-2}
RBMX	Rna binding motif protein, x chromosome	-1.09	9.05 x10^{-3}
DDX1	Dead box helicase 1	-1.03	4.38 x10^{-2}
PSMA2	Proteasome subunit alpha 2	1.01	4.50 x10^{-2}
WARS1		1.03	2.22 x10^{-3}
PFKP	Phosphofructokinase, platelet	1.07	4.42 x10^{-2}
NAMPT	Nicotinamide phosphoribosyltransferase	1.12	7.07 x10^{-3}
LAP3	Leucine aminopeptidase 3	1.14	2.79 x10^{-3}
CASP3	Caspase 3	1.15	1.35 x10^{-2}
EPRS1		1.22	8.17 x10^{-6}
GMFB	Glia maturation factor, beta	1.24	2.61 x10^{-3}
HADH	Hydroxyacyl-coenzyme a dehydrogenase	1.25	1.36 x10^{-3}
CNP	2',3'-cyclic nucleotide 3' phosphodiesterase	1.30	3.74 x10^{-4}
CMPK2	Cytidine/uridine monophosphate kinase 2	1.35	6.41 x10^{-5}
EIF3A	Eukaryotic translation initiation factor 3, subunit a	1.37	8.49 x10^{-4}
AP3M2	Adaptor-related protein complex 3, mu 2 subunit	1.38	1.03 x10^{-8}
HADHB		1.41	1.73 x10^{-8}
VAT1	Vesicle amine transport 1	1.51	1.59 x10^{-7}
SRSF2	Serine and arginine-rich splicing factor 2	1.52	1.82 x10^{-4}
CMTR1		1.73	9.23 x10^{-4}
B2M	Beta-2 microglobulin	1.87	9.13 x10^{-6}
IRGM1	Immunity-related gtpase family m member 1	2.44	2.06 x10^{-5}
STAT1		2.45	2.06 x10^{-10}
IFIT1	Interferon-induced protein with tetratricopeptide repeats 1	2.49	3.39 x10^{-6}
ISG15		2.68	1.02 x10^{-7}
BCLAF1	Bcl2-associated transcription factor 1	5.22	8.75 x10^{-6}
**JAK-STAT signalling pathway**			
STAT1	Signal transducer and activator of transcription 1	2.45	2.06 x10^{-10}

Gene symbol	Gene name	Proteomics	
		Fold change	q-value
**Interleukins**			
CNTFR	Ciliary neurotrophic factor receptor	1.34	2.96×10^{-3}
**RNA processing**			
DHX30	DExH-box helicase 30	1.88	4.99×10^{-5}
DDX39B	DEAD box helicase 39b	-1.07	2.69×10^{-2}
**Leukocytes**			
RAC2	Rac family small gtpase 2	3.32	1.45×10^{-5}
MSN	Moesin	2.06	3.40×10^{-9}
GFAP	Glial fibrillary acidic protein	3.89	1.54×10^{-6}

Supplementary Table 3 Full list of interferon stimulated genes (ISGs), chemokines, and cGAS-STING genes that were analysed for differential expression in genes and protein

Gene symbol	Gene name
**Interferon stimulated genes**	
Abtb2	Ankyrin repeat and BTB domain containing 2
Ac025171.1	
Adam9	ADAM metallopeptidase domain 9
Adar	Adenosine deaminase, RNA-specific
Agrn	Agrin
Aida	
Akr7a2	
Ankfy1	Ankyrin repeat and FYVE domain containing 1
Ap3m2	Adaptor-related protein complex 3, mu 2 subunit
Apobec3	Apolipoprotein B mrna editing enzyme, catalytic polypeptide 3
Apobec3g	
Apol1	
Apol2	
Apol3	
Apol4	
Apol6	Apolipoprotein L 6
Apol9a	Apolipoprotein L 9a
Apol9b	Apolipoprotein L 9b
Arap2	Arfgap with rhogap domain, ankyrin repeat and PH domain 2
Arid3a	AT-rich interaction domain 3A
Arid5b	AT-rich interaction domain 5B
Arl4a	ADP-ribosylation factor-like 4A
Atp10a	Atpase, class V, type 10A
Atp6v0a4	Atpase, H+ transporting, lysosomal V0 subunit A4
Atp6v0b	Atpase, H+ transporting, lysosomal V0 subunit B
Auts2	Autism susceptibility candidate 2
Avpr2	
B2m	Beta-2 microglobulin
Bag1	BCL2-associated athanogene 1
Bak1	BCL2-antagonist/killer 1
Bank1	B cell scaffold protein with ankyrin repeats 1
Batf2	Basic leucine zipper transcription factor, ATF-like 2
Bax	BCL2-associated X protein
Bbc3	BCL2 binding component 3
Bbx	Bobby sox HMG box containing
Bcl2	B cell leukemia/lymphoma 2
Bclaf1	BCL2-associated transcription factor 1
Bpgm	2,3-bisphosphoglycerate mutase
Brca1	Breast cancer 1, early onset
Bst2	Bone marrow stromal cell antigen 2

Gene symbol	Gene name
Btg1	BTG anti-proliferation factor 1
C1r	
C1s	
Cald1	Caldesmon 1
Calr	Calreticulin
Casp1	Caspase 1
Casp3	Caspase 3
Casp4	Caspase 4, apoptosis-related cysteine peptidase
Casp7	Caspase 7
Casp8	Caspase 8
Ccdc91	Coiled-coil domain containing 91
Ccl2	C-C motif chemokine ligand 2
Ccl5	C-C motif chemokine ligand 5
Ccl7	C-C motif chemokine ligand 7
Ccnyl1	
Ccrl2	C-C motif chemokine receptor-like 2
Cd164	CD164 antigen
Cd274	CD274 antigen
Cd38	CD38 antigen
Cd40	CD40 antigen
Cd47	CD47 antigen (Rh-related antigen, integrin-associated signal transducer)
Cd69	CD69 antigen
Cd74	CD74 antigen (invariant polypeptide of major histocompatibility complex, class II antigen-associated)
Cd80	CD80 antigen
Cd86	CD86 antigen
Cdk1	Cyclin dependent kinase 1
Cdkn1a	Cyclin dependent kinase inhibitor 1A
Cdkn1b	Cyclin dependent kinase inhibitor 1B
Ceacam1	CEA cell adhesion molecule 1
Cebpd	CCAAT/enhancer binding protein delta
Cfb	
Cfh	Complement component factor h
Cgas	
Ciita	Class II transactivator
Clic4	Chloride intracellular channel 4
Cmklr1	Chemerin chemokine-like receptor 1
Cmpk2	Cytidine/uridine monophosphate kinase 2
Cmtr1	
Cnp	2',3'-cyclic nucleotide 3' phosphodiesterase
Col16a1	Collagen, type XVI, alpha 1
Csf1	Colony stimulating factor 1 (macrophage)
Csf2rb	Colony stimulating factor 2 receptor, beta, low-affinity (granulocyte-macrophage)
Csrp3	Cysteine and glycine-rich protein 3
Ctr9	CTR9 homolog, Paf1/RNA polymerase II complex component

Gene symbol	Gene name
Cx3cl1	C-X3-C motif chemokine ligand 1
Cxcl10	C-X-C motif chemokine ligand 10
Cxcl11	Chemokine (C-X-C motif) ligand 11
Cxcl9	C-X-C motif chemokine ligand 9
Cyb561d2	Cytochrome b-561 domain containing 2
Cycs	
Cyp24a1	Cytochrome P450, family 24, subfamily a, polypeptide 1
Dck	Deoxycytidine kinase
Ddit4	DNA-damage-inducible transcript 4
Ddx1	DEAD box helicase 1
Ddx17	DEAD box helicase 17
Ddx21	Dexd box helicase 21
Ddx58	
Ddx60	Dexd/H box helicase 60
Dhx58	Dexh-box helicase 58
Dnajc2	Dnaj heat shock protein family (Hsp40) member C2
Dtx3	Deltex 3, E3 ubiquitin ligase
Dtx3l	Deltex 3-like, E3 ubiquitin ligase
E2f2	E2f transcription factor 2
Ehd4	EH-domain containing 4
Eif2ak2	Eukaryotic translation initiation factor 2-alpha kinase 2
Eif2b1	Eukaryotic translation initiation factor 2B, subunit alpha
Eif3a	Eukaryotic translation initiation factor 3, subunit A
Eif4e3	Eukaryotic translation initiation factor 4E member 3
Elf1	E74 like ETS transcription factor 1
Elk4	ELK4, member of ETS oncogene family
Eprs1	
Eps15	Epidermal growth factor receptor pathway substrate 15
Epsti1	Epithelial stromal interaction 1
Ets2	E26 avian leukemia oncogene 2, 3' domain
Etv7	
Fam111a	Family with sequence similarity 111, member A
Fam46a	
Fas	Fas cell surface death receptor
Fcgr1a	
Fgl2	Fibrinogen-like protein 2
Fosl1	Fos-like antigen 1
Fpr1	Formyl peptide receptor 1
Fubp1	Far upstream element (FUSE) binding protein 1
Gbp1	
Gbp2	Guanylate binding protein 2
Gbp3	Guanylate binding protein 3
Gbp4	Guanylate binding protein 4
Gbp5	Guanylate binding protein 5

Gene symbol	Gene name
Gbp6	
Gbp7	Guanylate binding protein 7
Gch1	GTP cyclohydrolase 1
Gimap7	
Gmfb	Glia maturation factor, beta
Gmpr	Guanosine monophosphate reductase
Golga4	Golgin A4
Golm1	Golgi membrane protein 1
Gpr18	
Gzma	Granzyme A
Hadh	Hydroxyacyl-Coenzyme A dehydrogenase
Hadhb	
Helz2	Helicase with zinc finger 2, transcriptional coactivator
Herc5	
Herc6	Hect domain and RLD 6
Hif1a	Hypoxia inducible factor 1, alpha subunit
Hk2	Hexokinase 2
Hla-a	
Hla-b	
Hla-c	
Hla-dma	
Hla-dpa1	
Hla-dqa1	
Hla-dqb1	
Hla-drb1	
Hla-drb3	
Hla-drb4	
Hla-drb5	
Hla-e	
Hla-f	
Hla-g	
Hpse	Heparanase
Hsd17b11	Hydroxysteroid (17-beta) dehydrogenase 11
Hsh2d	Hematopoietic SH2 domain containing
Hspe	
Icam1	Intercellular adhesion molecule 1
Ido1	Indoleamine 2,3-dioxygenase 1
Ifi16	
Ifi203	Interferon activated gene 203
Ifi204	Interferon activated gene 204
Ifi205	Interferon activated gene 205
Ifi27	Interferon, alpha-inducible protein 27
Ifi30	Interferon gamma inducible protein 30
Ifi35	Interferon-induced protein 35

Gene symbol	Gene name
Ifi44	Interferon-induced protein 44
Ifi44l	Interferon-induced protein 44 like
Ifi6	
Ifih1	Interferon induced with helicase C domain 1
Ifit1	Interferon-induced protein with tetratricopeptide repeats 1
Ifit2	Interferon-induced protein with tetratricopeptide repeats 2
Ifit3	
Ifit4	
Ifit5	
Ifitm1	Interferon induced transmembrane protein 1
Ifitm2	
Ifitm3	
Ifnar2	Interferon (alpha and beta) receptor 2
Il10ra	Interleukin 10 receptor, alpha
Il12rb2	Interleukin 12 receptor, beta 2
Il15	Interleukin 15
Il15ra	Interleukin 15 receptor, alpha chain
Il18bp	Interleukin 18 binding protein
Il1rn	Interleukin 1 receptor antagonist
Il23a	Interleukin 23, alpha subunit p19
Il2rb	Interleukin 2 receptor, beta chain
Il4r	
Il6	Interleukin 6
Il7	Interleukin 7
Iqgap1	IQ motif containing gtpase activating protein 1
Irf1	Interferon regulatory factor 1
Irf2	Interferon regulatory factor 2
Irf4	Interferon regulatory factor 4
Irf5	Interferon regulatory factor 5
Irf7	Interferon regulatory factor 7
Irf8	Interferon regulatory factor 8
Irf9	Interferon regulatory factor 9
Irgm1	Immunity-related gtpase family M member 1
Irgm2	
Isg15	
Isg20	Interferon-stimulated protein
Isoc1	Isochorismatase domain containing 1
Itga2	Integrin alpha 2
Itga4	Integrin alpha 4
Itgb7	Integrin beta 7
Jade2	Jade family PHD finger 2
Jak2	Janus kinase 2
Klk3	
Klrk1	Killer cell lectin-like receptor subfamily K, member 1

Gene symbol	Gene name
Kptn	Kaptin
Lamp3	Lysosomal-associated membrane protein 3
Lap3	Leucine aminopeptidase 3
Lat	Linker for activation of T cells
Lats2	Large tumor suppressor 2
Lcp2	Lymphocyte cytosolic protein 2
Lgals3bp	Lectin, galactoside-binding, soluble, 3 binding protein
Lgals9	Lectin, galactose binding, soluble 9
Lilrb1	
Linc01128	
Lipa	Lysosomal acid lipase A
Lipg	Lipase, endothelial
Lpar6	Lysophosphatidic acid receptor 6
Lrrc37a11p	
Ly6e	Lymphocyte antigen 6 family member E
Lysmd2	Lysm, putative peptidoglycan-binding, domain containing 2
Map1b	Microtubule-associated protein 1B
Map3k10	Mitogen-activated protein kinase kinase kinase 10
Map3k14	
Marchf1	
Max	Max protein
Mb21d1	
Mettl7b	
Mia3	MIA SH3 domain ER export factor 3
Mlkl	Mixed lineage kinase domain-like
Mmp25	Matrix metallopeptidase 25
Mov10	Mov10 RISC complex RNA helicase
Ms4a4a	Membrane-spanning 4-domains, subfamily A, member 4A
Ms4a6a	
Mt2a	
Mthfd2	Methylenetetrahydrofolate dehydrogenase (NAD+ dependent), methenyltetrahydrofolate cyclohydrolase
Mvb12a	Multivesicular body subunit 12A
Mvp	Major vault protein
Mx1	MX dynamin-like gtpase 1
Mx2	MX dynamin-like gtpase 2
Mxd4	Max dimerization protein 4
Myd88	Myeloid differentiation primary response gene 88
Myo1g	Myosin IG
Nampt	Nicotinamide phosphoribosyltransferase
Nap1l1	
Ncoa3	Nuclear receptor coactivator 3
Ncoa7	Nuclear receptor coactivator 7
Nell2	NEL-like 2
Nfkb1	

Gene symbol	Gene name
Nfkbia	Nuclear factor of kappa light polypeptide gene enhancer in B cells inhibitor, alpha
Nlrc5	NLR family, CARD domain containing 5
Nmi	N-myc (and STAT) interactor
Nod1	Nucleotide-binding oligomerization domain containing 1
Nos2	Nitric oxide synthase 2, inducible
Nr4a3	Nuclear receptor subfamily 4, group A, member 3
Nt5c3	5'-nucleotidase, cytosolic III
Nt5c3a	
Nub1	Negative regulator of ubiquitin-like proteins 1
Nup93	Nucleoporin 93
Nup98	Nucleoporin 98
Oas1	
Oas1b	2'-5' oligoadenylate synthetase 1B
Oas1g	2'-5' oligoadenylate synthetase 1G
Oas2	2'-5' oligoadenylate synthetase 2
Oas3	2'-5' oligoadenylate synthetase 3
Oasl	
Oasl2	2'-5' oligoadenylate synthetase-like 2
Ogfr	Opioid growth factor receptor
P2ry14	Purinergic receptor P2Y, G-protein coupled, 14
P2ry6	Pyrimidinergic receptor P2Y, G-protein coupled, 6
Pank2	Pantothenate kinase 2
Parp1	Poly (ADP-ribose) polymerase family, member 1
Parp11	Poly (ADP-ribose) polymerase family, member 11
Parp12	Poly (ADP-ribose) polymerase family, member 12
Parp14	Poly (ADP-ribose) polymerase family, member 14
Parp4	Poly (ADP-ribose) polymerase family, member 4
Parp9	Poly (ADP-ribose) polymerase family, member 9
Pcgf5	Polycomb group ring finger 5
Pde4b	Phosphodiesterase 4B, camp specific
Pdxk	Pyridoxal (pyridoxine, vitamin B6) kinase
Peli1	Pellino 1
Pfkp	Phosphofructokinase, platelet
Ph615	
Phb	
Phf11	
Pias4	Protein inhibitor of activated STAT 4
Pik3ap1	Phosphoinositide-3-kinase adaptor protein 1
Pim1	Proviral integration site 1
Pla1a	Phospholipase A1 member A
Pla2g4a	Phospholipase A2, group IVA (cytosolic, calcium-dependent)
Plaat4	
Plaur	Plasminogen activator, urokinase receptor
Plod2	Procollagen lysine, 2-oxoglutarate 5-dioxygenase 2

Gene symbol	Gene name
Plscr1	Phospholipid scramblase 1
Pmaip1	Phorbol-12-myristate-13-acetate-induced protein 1
Pml	Promyelocytic leukemia
Pnp	
Pnpt1	Polyribonucleotide nucleotidyltransferase 1
Polr2b	Polymerase (RNA) II (DNA directed) polypeptide B
Ppa1	Pyrophosphatase (inorganic) 1
Ppm1k	Protein phosphatase 1K (PP2C domain containing)
Ppp3ca	Protein phosphatase 3, catalytic subunit, alpha isoform
Ppp5c	Protein phosphatase 5, catalytic subunit
Praf2	
Prame	
Prkcq	Protein kinase C, theta
Procr	Protein C receptor, endothelial
Psma2	Proteasome subunit alpha 2
Psma3	
Psmb10	Proteasome (prosome, macropain) subunit, beta type 10
Psmb2	Proteasome (prosome, macropain) subunit, beta type 2
Psmb8	Proteasome (prosome, macropain) subunit, beta type 8 (large multifunctional peptidase 7)
Psmb9	Proteasome (prosome, macropain) subunit, beta type 9 (large multifunctional peptidase 2)
Psme1	Proteasome (prosome, macropain) activator subunit 1 (PA28 alpha)
Psme2	
Ptgs2	Prostaglandin-endoperoxide synthase 2
Ptpn1	Protein tyrosine phosphatase, non-receptor type 1
Ptpn11	Protein tyrosine phosphatase, non-receptor type 11
Ptpn2	Protein tyrosine phosphatase, non-receptor type 2
Ptpn6	Protein tyrosine phosphatase, non-receptor type 6
Pyhin1	
Rabl3	RAB, member RAS oncogene family-like 3
Rac3	Rac family small gtpase 3
Rapgef6	Rap guanine nucleotide exchange factor (GEF) 6
Rbbp4	Retinoblastoma binding protein 4, chromatin remodeling factor
Rbck1	Ranbp-type and C3HC4-type zinc finger containing 1
Rbm43	RNA binding motif protein 43
Rbmx	RNA binding motif protein, X chromosome
Rfc2	Replication factor C (activator 1) 2
Rhoc	Ras homolog family member C
Rin2	Ras and Rab interactor 2
Ripk1	Receptor (TNFRSF)-interacting serine-threonine kinase 1
Ripk2	Receptor (TNFRSF)-interacting serine-threonine kinase 2
Rnasel	Ribonuclease L (2', 5'-oligoisoadenylate synthetase-dependent)
Rnf213	Ring finger protein 213
Rnf31	Ring finger protein 31
Rrp8	Ribosomal RNA processing 8

Gene symbol	Gene name
Rsad2	Radical S-adenosyl methionine domain containing 2
Rtp4	Receptor transporter protein 4
Samd9	
Samd9l	Sterile alpha motif domain containing 9-like
Samhd1	SAM domain and HD domain, 1
Sdc3	Syndecan 3
Sdcbp	Syndecan binding protein
Sectm1	
Sell	Selectin, lymphocyte
Selp	Selectin, platelet
Sem1	
Septin4	
Serping1	Serine (or cysteine) peptidase inhibitor, clade G, member 1
Sf3a1	Splicing factor 3a, subunit 1
Skp1	
Slamf7	SLAM family member 7
Slc15a3	Solute carrier family 15, member 3
Slc25a28	Solute carrier family 25, member 28
Slc39a8	Solute carrier family 39 (metal ion transporter), member 8
Slfn5	Schlafen 5
Smad4	SMAD family member 4
Smchd1	SMC hinge domain containing 1
Socs1	
Socs3	Suppressor of cytokine signaling 3
Sod2	Superoxide dismutase 2, mitochondrial
Sp100	Nuclear antigen Sp100
Sp110	
Sppl2a	Signal peptide peptidase like 2A
Sri	Sorcin
Srp9	
Srsf2	Serine and arginine-rich splicing factor 2
Ssbp1	Single-stranded DNA binding protein 1
Ssbp3	Single-stranded DNA binding protein 3
Sspn	Sarcospan
St3gal5	ST3 beta-galactoside alpha-2,3-sialyltransferase 5
St8sia4	ST8 alpha-N-acetyl-neuraminide alpha-2,8-sialyltransferase 4
Stat1	
Stat2	Signal transducer and activator of transcription 2
Stat3	Signal transducer and activator of transcription 3
Stat4	Signal transducer and activator of transcription 4
Sun2	Sad1 and UNC84 domain containing 2
Tap1	Transporter 1, ATP-binding cassette, sub-family B (MDR/TAP)
Tapbp	TAP binding protein
Tdrd7	Tudor domain containing 7

Gene symbol	Gene name
Tead1	TEA domain family member 1
Tead4	TEA domain family member 4
Tent5a	
Tgtp1	
Tiparp	TCDD-inducible poly(ADP-ribose) polymerase
Tlk2	Tousled-like kinase 2 (Arabidopsis)
Tmem106a	Transmembrane protein 106A
Tmem140	
Tmem176a	Transmembrane protein 176A
Tnfaip2	Tumor necrosis factor, alpha-induced protein 2
Tnfaip3	Tumor necrosis factor, alpha-induced protein 3
Tnfaip6	Tumor necrosis factor alpha induced protein 6
Tnfsf10	Tumor necrosis factor (ligand) superfamily, member 10
Tnip3	TNFAIP3 interacting protein 3
Tor1b	Torsin family 1, member B
Tor3a	Torsin family 3, member A
Trafd1	TRAF type zinc finger domain containing 1
Trappc10	Trafficking protein particle complex 10
Trex1	
Trim14	Tripartite motif-containing 14
Trim21	Tripartite motif-containing 21
Trim22	
Trim25	Tripartite motif-containing 25
Trim26	Tripartite motif-containing 26
Trim30a	Tripartite motif-containing 30A
Trim5	Tripartite motif-containing 5
Ttyh2	Tweety family member 2
Tubb	
Txnip	Thioredoxin interacting protein
Uba7	Ubiquitin-like modifier activating enzyme 7
Ubd	Ubiquitin D
Ube2d3	Ubiquitin-conjugating enzyme E2D 3
Ube2l6	Ubiquitin-conjugating enzyme E2L 6
Ube2z	Ubiquitin-conjugating enzyme E2Z
Upp1	Uridine phosphorylase 1
Usp18	Ubiquitin specific peptidase 18
Usp25	Ubiquitin specific peptidase 25
Vamp5	Vesicle-associated membrane protein 5
Vamp8	Vesicle-associated membrane protein 8
Vat1	Vesicle amine transport 1
Vcam1	Vascular cell adhesion molecule 1
Vcpip1	Valosin containing protein (p97)/p47 complex interacting protein 1
Vegfc	Vascular endothelial growth factor C
Wars1	

Gene symbol	Gene name
Xaf1	XIAP associated factor 1
Xcl1	Chemokine (C motif) ligand 1
Xrcc6	X-ray repair complementing defective repair in Chinese hamster cells 6
Xrn1	5'-3' exoribonuclease 1
Zbp1	Z-DNA binding protein 1
Zc3hav1	Zinc finger CCCH type, antiviral 1
Zcchc2	Zinc finger, CCHC domain containing 2
Zfp36l2	Zinc finger protein 36, C3H type-like 2
Znfx1	Zinc finger, NFX1-type containing 1
Chemokines	
Ccl1	C-C motif chemokine ligand 1
Ccl2	C-C motif chemokine ligand 2
Ccl3	C-C motif chemokine ligand 3
Ccl4	C-C motif chemokine ligand 4
Ccl5	C-C motif chemokine ligand 5
Ccl6	C-C motif chemokine ligand 6
Ccl7	C-C motif chemokine ligand 7
Ccl8	C-C motif chemokine ligand 8
Ccl9	C-C motif chemokine ligand 9
Ccl11	C-C motif chemokine ligand 11
Ccl12	C-C motif chemokine ligand 12
Ccl17	C-C motif chemokine ligand 17
Ccl19	C-C motif chemokine ligand 19
Ccl20	C-C motif chemokine ligand 20
Ccl21a	C-C motif chemokine ligand 21 (serine)
Ccl22	C-C motif chemokine ligand 22
Ccl24	C-C motif chemokine ligand 24
Ccl25	C-C motif chemokine ligand 25
Ccl26	C-C motif chemokine ligand 26
Ccl28	C-C motif chemokine ligand 28
Cx3cl1	C-X3-C motif chemokine ligand 1
Cxcl1	C-X-C motif chemokine ligand 1
Cxcl2	C-X-C motif chemokine ligand 2
Cxcl3	C-X-C motif chemokine ligand 3
Cxcl5	C-X-C motif chemokine ligand 5
Cxcl9	C-X-C motif chemokine ligand 9
Cxcl10	C-X-C motif chemokine ligand 10
Cxcl11	C-X-C motif chemokine ligand 11
Cxcl12	C-X-C motif chemokine ligand 12
Cxcl13	C-X-C motif chemokine ligand 13
Cxcl14	C-X-C motif chemokine ligand 14
Cxcl15	C-X-C motif chemokine ligand 15
**cGAS-STING**	
Mb21d1	Mab-21 domain-containing protein 1 (Cgas)

Gene symbol	Gene name
Tbk1	Tank-binding kinase 1
Tmem173	Stimulator of interferon genes (STING)
Irf3	Interferon regulatory factor 3
**Leukocyte adhesion**	
Cd11b	
Cd18	
Cd29	
Cd44	CD44 antigen
Cd49d	
Cd99	
Esl1	
Esm1	endothelial cell-specific molecule 1
Ic3b	
Icam1	intercellular adhesion molecule 1
Icam2	intercellular adhesion molecule 2
Icam3	
Icam4	
Icam5	intercellular adhesion molecule 5, telencephalin
Itgad	integrin, alpha D
Itgal	integrin alpha L
Itgam	integrin alpha M
Itgax	integrin alpha X
Itgb2	integrin beta 2
Jam1	
Jama	
Jamc	
Lfa1	
Lpam1	
Pecam1	platelet/endothelial cell adhesion molecule 1
Psgl1	
Rage	
Sele	selectin, endothelial cell
Vcam1	vascular cell adhesion molecule 1
Vla4	

Supplementary Table 4 Overview of genes from the reference list

Description	Number of genes
Genes in reference list	23 930
Reference list genes that mapped to Entrezgene IDs	22 537
Entrez IDs from reference list that are annotated in KEGG pathways	7321

Supplementary Table 5 Explanation of different terms and calculations for ORA by using Epstein-Barr virus infection as an example

**Description**	**Gene set size**	**Overlap**	**Expect**	**Enrichment ratio**
Epstein-Barr virus infection (mmu05169)	201	23	4.75	4.84

**Term**	**Description**	**Calculations for Epstein-Barr virus infection**
**Gene set size**	The number of genes that are in the reference list (background) and in the specific pathway	201
**Overlap**	The number of significant genes that are in the specific pathway	23
**Expected**	The gene set size divided by the number of reference genes that are annotated in the KEGG pathway through an entrez ID, multiplied by the number of analyte genes in the KEGG pathway	= 201 / 7321 * 173<br>= 4.749
	Total number of reference genes	23 930
	Reference genes mapped to enterez id	22 537
	Entrez IDs from reference list that are annotated in KEGG pathways	7321
	Analysed genes that are mapped to an entrez ID and that are in the KEGG database	173
**Enrichment ratio**	The number of significant genes divided by the expected	= 23 / 4.749<br>= 4.843
